# SeOMLR: one-step multi-view latent representation with self-weighted ensemble learning for multi-omics cancer subtyping

**DOI:** 10.1093/bioinformatics/btag074

**Published:** 2026-03-05

**Authors:** Wenjing Song, Yesen Sun, Le Ou-Yang

**Affiliations:** School of Science, Southwest Petroleum University, Chengdu, 610500, China; School of Arts and Sciences, Guangzhou Maritime University, Guangzhou, 510725, China; SMBU-MSU-BIT Joint Laboratory on Bioinformatics and Engineering Biology, Faculty of Engineering, Shenzhen MSU-BIT University, Shenzhen, 518172, China

## Abstract

**Motivation:**

Accurate cancer subtyping is critically important for cancer treatment due to significant molecular heterogeneity. While existing methods with multi-omics integration have achieved some success in cancer subtype identification by leveraging the rich information provided by multi-omics data, most approaches remain limited by an overemphasis on cross-omics consistency at the expense of intra-omics specificity. Furthermore, a two-step scheme is often adopted to extract cluster structure from a consistency matrix or a continuous indicator matrix by k-means, which inevitably leads to information loss and unstable clusters.

**Results:**

To overcome these issues, we propose seOMLR, a one-step multi-view latent representation method with self-weighted ensemble learning for cancer subtyping. Using relaxed exclusivity constraints and consistency regularization terms, seOMLR exploits the specificity and consistency of multi-omics data by building a sparse low-rank self-representation framework. Simultaneously, a self-weighted ensemble strategy is introduced to adaptively incorporate prior subtyping information from other methods, indirectly promoting specificity and consistency learning. Moreover, the discrete clustering structure is subsequently extracted via spectral rotation to avoid information loss and cluster instability. Through joint iterative optimization of fusion and clustering, seOMLR enhances subtyping accuracy. Experiments on both simulated datasets and eight real multi-omics cancer datasets from TCGA demonstrate that seOMLR outperforms competing methods, achieving efficient multi-omics data fusion and providing computational framework support for cancer subtyping research.

**Availability and implementation:**

Supplementary data are available at *Bioinformatics* online.

## 1. Introduction

Cancer is a complex and multifactorial disease whose heterogeneity poses major challenges to precision medicine, driving the need for more refined cancer subtyping based on molecular characteristics and clinical behavior. In-depth analysis of omics data helps to unravel the complex mechanisms underlying cancer heterogeneity (Ma & Gao 2012).

The rapid development of next-generation sequencing technologies has enabled the generation of large-scale omics data from large projects such as The Cancer Genome Atlas (TCGA) (Cancer et al. 2013), which provides heterogeneous data on the same sample for more than 33 cancers, creating unprecedented opportunities for cancer subtyping research. Many cancer subtyping studies primarily relied on single data type (Ma et al. 2017), however, a human genome is complex and regulated at multiple levels. Independent analysis of single data cannot yield systematic insights into organisms involving intricate regulatory processes (Kitano 2002).

Machine learning methods provide a new perspective for cancer subtyping by integrating and analysing multi-omics data (Rappoport & Shamir 2018). Early multi-omics studies for identifying cancer subtypes mainly focused on simple splicing (Wang et al. 2013, Nguyen et al. 2019) or statistical modeling (Shen et al. 2010, Mo et al. 2013, 2018). For example, LRAcluster (Wang et al. 2013) concatenates multiple heterogeneous omics data by probabilistically modeling the distribution of numerical, count, and discrete features, but this integration method is prone to cause dimensionality issues and redundant feature interference despite its simplicity and efficiency. The iCluster (Shen et al. 2010) method has pioneered a statistical integration paradigm based on joint latent variable models, mapping multi-omics data to a low dimensional latent space through probability distribution assumptions. Nevertheless, such probability-based statistical models are sensitive to data distribution assumptions, posing risks of model mismatch in practical applications.

Similarity-based methods (Wang et al. 2014, Rappoport & Shamir 2019, Duan et al. 2024, Miao et al. 2025, Jessica et al. 2025) are widely used in cancer subtyping research, whose core idea is to build a multi-omics network based on the similarity between samples and generate a consistent network through a fusion strategy for clustering. PartIES (Miao et al. 2025) proposes a partition-level integration method based on diffusion-enhanced similarity identification, which enhances the robustness by integrating similarity matrices through diffusion strategies and iterative optimization.

Recently, deep learning models utilize deep neural networks to extract nonlinear representations of multi-omics data for cancer subtyping (Wu et al. 2024, Yang et al. 2025, Zhang et al. 2025). For example, ADFusion (Zhang et al. 2025) innovatively adopts a layered graph convolution framework to construct high-quality representations of multimodal cancer data, and realizes multimodal dynamic fusion through the deep equilibrium theory. Although deep learning–based algorithms have demonstrated strong empirical performance in subtype discovery, they typically require large-scale training data and careful regularization. In high-dimensional yet small-sample cancer scenarios, overfitting can be a practical concern, and subtype solutions may vary with model specification and training strategy. These considerations motivate optimization-driven multi-view integration frameworks with explicit structural regularization.

Some multi-view subspace clustering algorithms (Yang et al. 2022, Shi et al. 2023, Tian et al. 2024) have become essential tools for cancer subtyping, which treat multi-omics data as multiple views and perform joint optimization through strategies such as low-rank constraints, graph fusion, or regularization. SMCC (Tian et al. 2024) integrates the low-rank subspace representation and entropy to fuse networks, and minimizes the distributional differences between the similarity networks and the fusion network by co-regularization.

Ensemble learning approaches (Mitra & Saha 2019, Song et al. 2022) integrate clustering results from multiple algorithms or data sources, effectively mitigating single model bias and synergistically leveraging complementary subtyping information to generate robust classifications. The subtype-WESLR (Song et al. 2022) method constructs a common latent subspace, while maintaining local structural consistency and base cluster consistency derived from various clustering methods, thereby improving the generalization through ensemble learning and iterative optimization.

**Figure 1:**
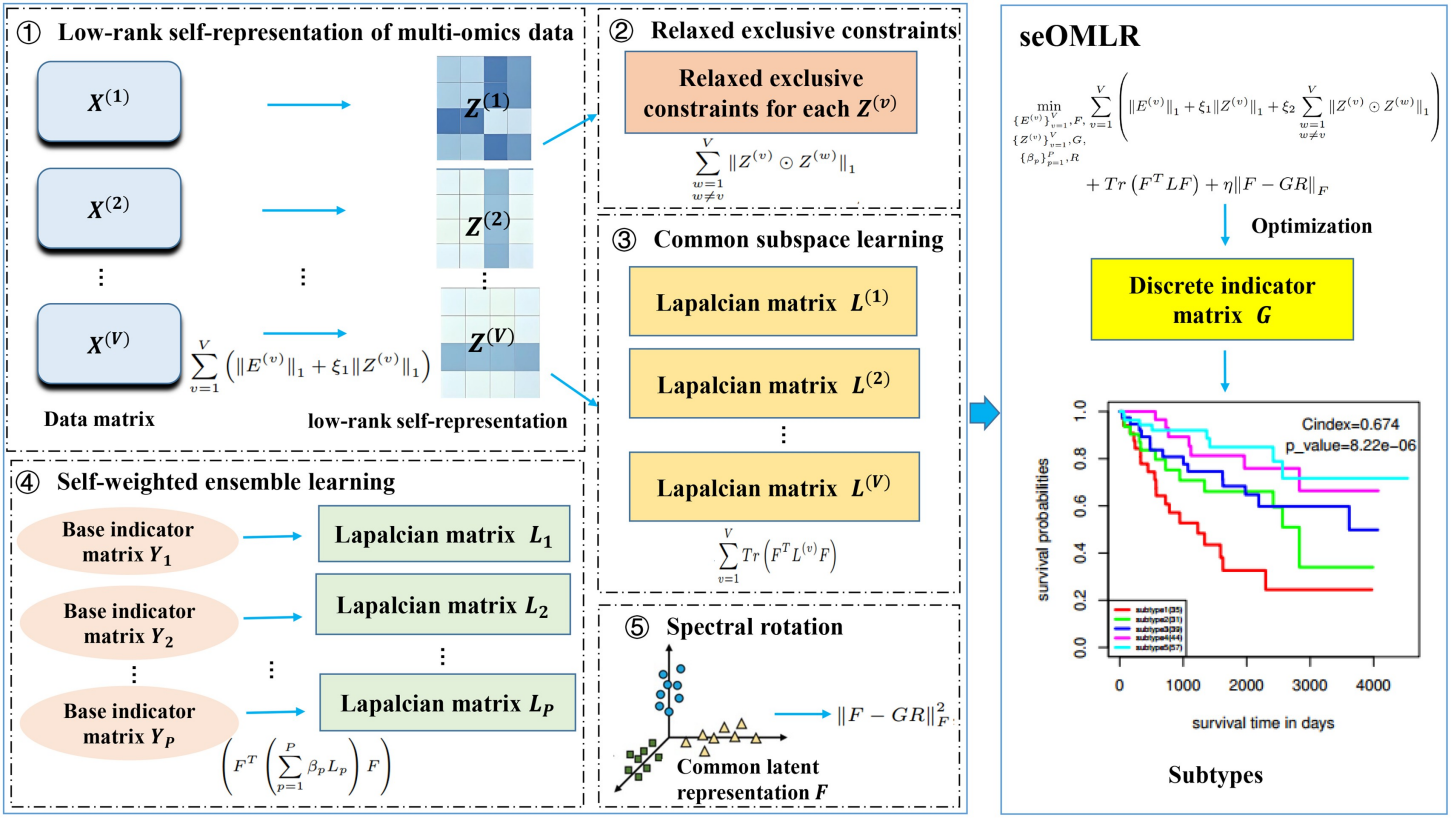
Workflow of seOMLR. Matrices $X^{\left(v\right)}$  (v=1,2,…,V) and $Y_{p}$  (p=1,2,…,P) are the inputs. $Z^{\left(v\right)}$ is the self-representation matrix of $X^{\left(v\right)}$. SeOMLR builds a sparse low-rank self-representation framework to exploit intra-omics specificity and cross-omics consistency of multi-omics data via introducing relaxed exclusivity constraints and consistent regularization. Meanwhile, a self-weighted ensemble strategy without additional parameters is designed to utilize subtyping information from other methods, indirectly promoting specificity and consistency learning. Besides, spectral rotation is applied to extract stable clusters structure of *F*, and to build a unified joint optimization framework for integration and clustering of multi-omics data.

Despite significant advances in various machine learning algorithms, most existing methods such as SNF and PartIES typically adopt a two-step scheme, which firstly obtains a spectral embedding matrix through graph fusion or a continuous indicator matrix, and then uses the k-means (Ding & He 2004) algorithm to cluster it to extract the discrete cluster structure. This inevitably causes information loss, resulting in unstable and suboptimal cluster results. Furthermore, some work like PartIES and subtype-WESLR may only focus on cross-view consistency while neglecting intra-view specificity during integration, which also decreases the clustering performance. To address these limitations, we propose a one-step multi-view latent representation method with self-weighted ensemble learning, termed seOMLR (Fig. 1), for cancer subtype identification. Our model constructs a sparse low-rank self-representation framework to exploit multi-omics data, in which specificity and consistency information are collaboratively mined by introducing relaxed exclusivity constraints and consistency regularization, respectively. Meanwhile, a parameter-free ensemble weighting strategy is designed to effectively incorporate subtyping information from other methods, indirectly promoting specificity and consistency learning. Moreover, we adopt spectral rotation to extract the discrete clustering structure to avoid information loss and instability. Ultimately, we achieve joint iterative optimization of fusion and clustering, enhancing seOMLR’s capability to integrate and classify multi-omics data. To validate the effectiveness of seOMLR, we conducted experiments on simulated datasets and eight publicly available multi-omics datasets from TCGA. Experimental results demonstrate that seOMLR outperforms some state-of-the-art competing methods, achieving efficient multi-omics data fusion and providing a powerful computational framework for cancer subtyping research.

## 2. Methods

Our proposed framework comprises five key components: low-rank self-representation of multi-omics data, relaxed exclusivity constraints, consistency regularization, self-weighted ensemble learning, and spectral rotation (Fig. 1). These modules are jointly optimized through an iterative optimization strategy to achieve collaborative enhancement of integration and clustering performance. The detailed methodology is described as follows.

### 2.1. Problem formalization

#### 2.1.1. Low-rank self-representation of multi-omics data

Suppose we have *n* samples (e.g. patients) and *V* views (e.g. miRNA, mRNA, and DNA methylation). The *v*-th view data is denoted as a matrix $X^{\left(v\right)}\in R^{d_{v}\times n}$  (v=1,2,…,V), where $d_{v}$ is the number of features in the *v*-th view.

For each feature matrix $X^{\left(v\right)}$, the sparse self-representation can be obtained by solving the following optimization problem:


(1) $$\begin{array}{cc} \;\underset{E^{\left(v\right)},Z^{\left(v\right)}}{\mathrm{arg min}}{||E^{\left(v\right)}||}_{1}+\xi_{1}{||Z^{\left(v\right)}||}_{1} & \;\\ s.t.\;X^{\left(v\right)}=X^{\left(v\right)}Z^{\left(v\right)}+E^{\left(v\right)},\mathrm{diag}(Z^{\left(v\right)})=0,v=1,\ldots ,V,\; \end{array}$$

where $X^{\left(v\right)}=X^{\left(v\right)}Z^{\left(v\right)}+E^{\left(v\right)}$ is the self-representation model. $Z^{\left(v\right)}\in R^{n\times n}$ and $E^{\left(v\right)}\in R^{n\times n}$ separately denote the self-representation matrix and error matrix of the *v*-th view. $\|\cdot {\|}_{1}$ is the $l_{1}$-norm for pursuing sparsity. The norm of error term $E^{\left(v\right)}$ depends on prior knowledge of noise or damage patterns. Our work employs only the $l_{1}$-norm to address sparse damage and representation by $\|E^{\left(v\right)}{\|}_{1}$ and $\|Z^{\left(v\right)}{\|}_{1}$, respectively. $\xi_{1}>0$ is a regularization parameter. The constraint $\mathrm{diag}(Z^{\left(v\right)})=0$ is used to exclude trivial solutions where $Z^{\left(v\right)}$ is the identity matrix.

Without loss of generality, extending the problem (1) into a multi-view case leads to the following objective function:


(2) $$\begin{array}{cc} \;\underset{{\left\{E^{\left(v\right)}\right\}}_{v=1}^{V},{\left\{Z^{\left(v\right)}\right\}}_{v=1}^{V}}{\arg\min}\sum_{v=1}^{V}\left(\|E^{\left(v\right)}{\|}_{1}+\xi_{1}\|Z^{\left(v\right)}{\|}_{1}\right)\\ & \begin{array}{c} s.t.\;X^{\left(v\right)}=X^{\left(v\right)}Z^{\left(v\right)}+E^{\left(v\right)},\mathrm{diag}(Z^{\left(v\right)})=0,v=1,\ldots ,V.\; \end{array} \end{array}$$

By resolving the problem (2), we can learn an underlying low-dimensional sparse representation for each data type.

#### 2.1.2. Relaxed exclusivity constraints

Tumor heterogeneity is manifested by multidimensional differences in genotype, phenotype, and so on, which directly lead to specific signals in different omics data. These specific pieces of information play a crucial role in identifying cancer subtypes. It is necessary to take them into account during multi-omics data integration for cancer subtyping. Here, we introduce relaxed exclusivity constraints to balance these specific pieces of information from different omics. For matrices $U\in R^{n\times n}$ and $W\in R^{n\times n}$, the ExRM (Guo et al. 2017) method defines the relaxed exclusivity that encourages *U* and *W* to be as diverse as possible with the Hadamard product and $l_{1}$-norm.

Definition 1 **(Relaxed Exclusivity (** Guo et al. 2017 **))**.

*Relaxed exclusivity between two matrices* $U\in R^{n\times n}$  *and* $W\in R^{n\times n}$  *is defined as* $D(U,W)=\|U⊙W{\|}_{1}$*, where* ⊙  *is the Hadamard product.*

Similar to ECMSC (Wang et al. 2017), we enforce the representations of different views to be as exclusive as possible by employing the relaxed exclusivity term which can be seamlessly incorporated with the low-rank self-representation framework of multi-omics data. The objective function (2) with relaxed exclusivity constraints can be written as


(3) $$\begin{array}{c} \underset{{\left\{E^{\left(v\right)},Z^{\left(v\right)}\right\}}_{v=1}^{V}}{\arg\min}\sum_{v=1}^{V}\left({||E^{\left(v\right)}||}_{1}+\xi_{1}{||Z^{\left(v\right)}||}_{1}+\xi_{2}\sum_{\begin{array}{c} w=1\\ w\neq v \end{array}}^{V}\|Z^{\left(v\right)}⊙Z^{\left(w\right)}{\|}_{1}\right)\\ s.t.\;X^{\left(v\right)}=X^{\left(v\right)}Z^{\left(v\right)}+E^{\left(v\right)},\mathrm{diag}\left(Z^{\left(v\right)}\right)=0,v=1,\ldots ,V, \end{array}$$

where $\xi_{2}>0$ is a regularization parameter.

#### 2.1.3. Consistency regularization

After obtaining the sparse self-representation $Z^{\left(v\right)}(v=1,2,\ldots ,V)$ from the objective function (3), we employ Laplacian regularization to extract local cluster structure from $Z^{\left(v\right)}$. Suppose $S^{\left(v\right)}=\frac{Z^{\left(v\right)}+|Z^{\left(v\right)}{\;}^{T}|}{2}$, the Laplacian matrix of $Z^{\left(v\right)}$ can be written as


(4) $$L^{\left(v\right)}=D^{\left(v\right)}-S^{\left(v\right)}\;v=1,\ldots ,V,$$

where $D^{\left(v\right)}\in R^{n\times n}$ is a diagonal matrix with $D^{\left(v\right)}(i,i)=\sum_{j=1}^{n}S^{\left(v\right)}(i,j)$.

The consistency information among multi-omics data is extracted by multi-view Laplacian regularization, i.e.


(5) $$\begin{array}{cc} & \underset{F}{\arg\min}\sum_{v=1}^{V}Tr\left(F^{T}L^{\left(v\right)}F\right)\\ & \begin{array}{c} s.t.\;F^{T}F=I,F>0,\; \end{array} \end{array}$$

where $F\in R^{n\times c}$ is an indicator matrix with orthogonality and *c* is the number of clusters. Besides, *I* is an identity matrix.

#### 2.1.4. Self-weighted ensemble learning

Integrating the subtyping information of different clustering methods with an ensemble strategy can effectively promote multi-omics integration for identifying cancer subtypes, which has been demonstrated in our previous work subtype-WESLR. However, it is necessary to adjust the parameters to achieve ensemble learning in subtype-WESLR. Here, a self-weighted ensemble learning strategy will be designed without introducing additional parameters in seOMLR.

Similarly, we employ the Laplacian regularization to make the learned *F* maintain the local consistency of base clustering results (e.g. iClusterPlus, SNF, mocluster (Meng et al. 2016)), whose indicator matrix $Y_{p}\in {\left\{0,1\right\}}^{n\times c_{p}}$ (p=1,2,…,P) is generated by the *p*-th base clustering algorithm in which $c_{p}$ and *P* are the number of clusters in the *p*-th base algorithm and the number of base algorithms, respectively. For each indicator matrix, a graph model $S_{p}\in {\left\{0,1\right\}}^{n\times n}$ is constructed with $S_{p}=Y_{p}Y_{p}^{T}$ where $S_{p}(i,j)=1$ indicates that samples *i* and *j* belong to the same cluster under the *p*-th base clustering algorithm and 0 otherwise. The graph Laplacian matrix $L_{p}$ can be computed by $L_{p}=D_{p}-S_{p}$, in which $D_{p}$ is a diagonal matrix with $D_{p}(i,i)=\sum_{j=1}^{n}S_{p}(i,j)$. The effective information of basic clustering can be reflected in the graph Laplacian matrix, which is applied to ensemble learning to adaptively optimize the subspace *F*. Base indicator consistency of distinct clustering for *F* can be obtained by


(6) $$\begin{array}{cc} & \underset{F}{\arg\min}Tr\left(F^{T}\left(\sum_{p=1}^{P}\beta_{p}L_{p}\right)F\right)\\ & \begin{array}{c} s.t.\;F^{T}F=I,F>0,\; \end{array} \end{array}$$

where the weight coefficient $\beta_{p}$ balances the contribution of the *p*-th base clustering method to the prediction and is given by


(7) $$\begin{array}{cc} & \beta_{p}=\frac{1}{2\sqrt{Tr\left(F^{T}L_{p}F\right)}}\;(p=1,\ldots ,P), \end{array}$$

whose derivation process is detailed in the supplementary material. As shown in equation (7), this formulation ensures that all base clustering methods contribute to subtype identification without introducing extra parameters.

Supposing that *F* can be calculated by (6) when $\beta_{p}$ is fixed, this *F* will be continuously used to update $\beta_{p}$ according to (7), which inspires us to take an alternating optimization strategy to compute *F* and $\beta_{p}$ iteratively.

#### 2.1.5. Spectral rotation

Upon obtaining the continuity indication matrix *F*, the common practice involving applying k-means clustering to it may deviate significantly from the true discrete solution, thereby compromising the final clustering accuracy. Furthermore, the two-step strategy that separates clustering from integration may result in some loss of information. Hence, we employ the spectral rotation technique (Huang et al. 2013) to merge clustering and integration within a single optimization framework. The objective function for applying spectral rotation to *F* is


(8) $$\begin{array}{cc} & \underset{G,R}{\arg\min}{||F-GR||}_{F}^{2}\\ & s.t.\;R^{T}R=I,\;G\in \mathrm{Ind},\;G\in {\left\{0,1\right\}}^{n\times c}, \end{array}$$

where G∈Ind denotes *G* is an indicator matrix of which the unique 1 in each row vector indicates its cluster membership, and $R^{T}R=I$ means the normalized orthonormal constraint imposed on the c×c matrix *R*, which guarantees that *G* best approximates *FR* among all discrete cluster membership indicator matrices.

Let $L=\xi_{3}\left(\sum_{v=1}^{V}L^{\left(v\right)}+\delta \sum_{p=1}^{P}\beta_{p}L_{p}\right)$. Combining (3), (5), (6), and (8), we can write seOMLR as


$$\underset{\begin{array}{c} {\left\{E^{\left(v\right)}\right\}}_{v=1}^{V},F,\\ {\left\{Z^{\left(v\right)}\right\}}_{v=1}^{V},G,\\ {}[0.2em]{\left\{\beta_{p}\right\}}_{p=1}^{P},R \end{array}}{\min}\sum_{v=1}^{V}\left({||E^{\left(v\right)}||}_{1}+\xi_{1}{||Z^{\left(v\right)}||}_{1}+\xi_{2}\sum_{\begin{array}{c} w=1\\ w\neq v \end{array}}^{V}\|Z^{\left(v\right)}⊙Z^{\left(w\right)}{\|}_{1}\right)$$

(9) $$\begin{array}{c} +Tr\left(F^{T}LF\right)+\eta {||F-GR||}_{F}^{2},\\ s.t.\\ \;X^{\left(v\right)}=X^{\left(v\right)}Z^{\left(v\right)}+E^{\left(v\right)},\\ \mathrm{diag}\left(Z^{\left(v\right)}\right)=0,\;v=1,\ldots ,V,\\ F^{T}F=I,F>0,\;R^{T}R=I,\\ G\in \mathrm{Ind},\;G\in {\left\{0,1\right\}}^{n\times c}, \end{array}$$

where $\xi_{3}>0$ and η>0 are regularization parameters. δ≥0 is used to balance feature matrices and base ensemble clustering results. By solving (9), seOMLR can learn a shared latent representation across multiple omics, while preserving the unique characteristics of each omic. It also leverages subtyping information from other clustering methods to achieve stable and discrete clustering results.

### 2.2. Optimization

We optimize the objective function (9) by alternately and iteratively updating to obtain the solutions, as analysed in the supplementary material, in which we optimize the value of $\beta_{p}$, $E^{\left(v\right)}$, and $Z^{\left(v\right)}$ given *F*; and then employ them to update *F*, *R*, and *G*, i.e.:

**Step 1: update**  $\beta_{p}$, $E^{\left(v\right)}$**, and**  $Z^{\left(v\right)}$. When *F*, *R*, and *G* are fixed, we update $\beta_{p}$ by (7). Meanwhile, the objective function (9) is written with respect to $E^{\left(v\right)}$ and $Z^{\left(v\right)}$ as


(10) $$\begin{array}{c} \underset{{\left\{E^{\left(v\right)},Z^{\left(v\right)}\right\}}_{v=1}^{V}}{\arg\min}\sum_{v=1}^{V}({||E^{\left(v\right)}||}_{1}+\xi_{1}{||Z^{\left(v\right)}||}_{1}+\xi_{2}\sum_{\begin{array}{c} w=1\\ w\neq v \end{array}}^{V}\|Z^{\left(v\right)}⊙Z^{\left(w\right)}{\|}_{1}\\ +Tr\left(F^{T}L^{\left(v\right)}F\right))\\ s.t.\;X^{\left(v\right)}=X^{\left(v\right)}Z^{\left(v\right)}+E^{\left(v\right)},\mathrm{diag}\left(Z^{\left(v\right)}\right)=0,v=1,\ldots ,V, \end{array}$$

Algorithm 1 ADMM for solving $E^{\left(v\right)}$ and $Z^{\left(v\right)}$

**Input:** multi-view feature matrices ${\left\{X^{\left(v\right)}\right\}}_{v=1}^{V}$; indicator matrix *F*; parameters $\xi_{1}$, $\xi_{2}$, $\xi_{3}$, ρ=1.2, and $\epsilon =2\times 10^{-4}$; maximum iterations $T_{1}$ for ADMM innerloop.

**Output:** error matrices ${\left\{E^{\left(v\right)}\right\}}_{v=1}^{V}$; self-representation matrices ${\left\{Z^{\left(v\right)}\right\}}_{v=1}^{V}$.

1: Compute *D* with elements $D_{i,j}=\sum_{i,j=1}^{n}\frac{1}{2}\|f^{i}-f^{j}{\|}_{F}^{2}$;

2: **repeat** 

3:  Update $Z^{\left(v\right)}$ by (11);

4:  Update $E^{\left(v\right)}$ by (12);

5:  Update $C^{\left(v\right)}$ by (13);

6:  Update $Q_{1}$ and $Q_{2}$ by (14);

7:  Update μ=μρ;

8: **until** convergence $\|X^{\left(v\right)}-X^{\left(v\right)}C^{\left(v\right)}-E^{\left(v\right)}{\|}_{\infty }<\epsilon$ or reach the maximum iterations $T_{1}$.

Similar to ECMSC, we solve for $E^{\left(v\right)}$ and $Z^{\left(v\right)}$ of (10) separately for each *v* (v=1,…,V) by using the Alternating Direction Method of Multipliers (ADMM) (Lin et al. 2011). The optimization process is analysed in the supplementary material and summarized in Algorithm 1, and the updated iteration formulas are as follows


(11) $$\{\begin{array}{c} {\hat{Z}}^{\left(v\right)}=S_{\frac{1}{\mu }(\xi_{1}E+\xi_{2}\sum_{w=1,w\neq v}^{V}Z^{\left(w\right)}+\xi_{3}D)}\left[C^{\left(v\right)}+\frac{Q_{2}}{\mu }\right]\\ Z^{\left(v\right)}={\hat{Z}}^{\left(v\right)}-\mathrm{diag}({\hat{Z}}^{\left(v\right)}), \end{array}$$

(12) $$\begin{array}{c} E^{\left(v\right)}=S_{\frac{1}{\mu }}\left[X^{\left(v\right)}-X^{\left(v\right)}C^{\left(v\right)}+\frac{Q_{1}}{\mu }\right], \end{array}$$

(13) $$\begin{array}{cc} C^{\left(v\right)}={\left(X^{\left(v\right)}{\;}^{T}X^{\left(v\right)}+I\right)}^{-1}[X^{\left(v\right)}{\;}^{T}\left(X^{\left(v\right)}-E^{\left(v\right)}+\frac{Q_{1}}{\mu }\right) & \\ +Z^{\left(v\right)}-\mathrm{diag}\left(Z^{\left(v\right)}\right)-\frac{Q_{2}}{\mu }], & \end{array}$$

(14) $$\begin{array}{c} Q_{1}=Q_{1}+\mu \left(X^{\left(v\right)}-X^{\left(v\right)}C^{\left(v\right)}-E^{\left(v\right)}\right),\\ Q_{2}=Q_{2}+\mu \left(C^{\left(v\right)}-Z^{\left(v\right)}+\mathrm{diag}(Z^{\left(v\right)}\right). \end{array}$$

where $S_{\tau }[\cdot ]$ and $E\in R^{n\times n}$ in (11) are the shrinkage thresholding operator and a matrix with all elements equal to 1, respectively, and $C^{\left(v\right)}$, $Q_{1}$, and $Q_{2}$ are intermediate variables introduced to solve for $E^{\left(v\right)}$ and $Z^{\left(v\right)}$. *D* is a n×n matrix with the element $D_{i,j}=\sum_{i,j=1}^{n}\frac{1}{2}\|f^{i}-f^{j}{\|}_{F}^{2}$, of which $f^{i}$ is the *i*-th row vector of *F*. Besides, μ is a positive penalty scalar.

**Step 2: update *F*, *R*, and *G***. Fixed $Z^{\left(v\right)}$, $E^{\left(v\right)}$, and $\beta_{p}$, (9) becomes as in relation to *F*, *R*, and *G*, i.e.


(15) $$\begin{array}{cc} \underset{F,G,R}{\arg\min} & Tr\left(F^{T}LF\right)+\eta {||F-GR||}_{F}^{2},\\ & \begin{array}{c} s.t.\\ F^{T}F=I,F>0,\;R^{T}R=I,\\ G\in \mathrm{Ind},\;G\in {\left\{0,1\right\}}^{n\times c}, \end{array} \end{array}$$

Algorithm 2 solving for *F*, *R*, and *G*

**Input:** self-representation matrices ${\left\{Z^{\left(v\right)}\right\}}_{v=1}^{V}$; base clustering matrices ${\left\{Y_{p}\right\}}_{p=1}^{P}$; parameters $\xi_{3}$, δ, η, σ, and $\epsilon =2\times 10^{-4}$; maximum iterations $T_{2}$ for spectral rotation.

**Output:** cluster indicator matrix *G*; orthonormal matrix *R*; indicator matrix *F*.

1: Compute Laplacian matrix ${\left\{L^{\left(v\right)}\right\}}_{v=1}^{V}$ and ${\left\{L_{p}\right\}}_{p=1}^{P}$ based on ${\left\{Z^{\left(v\right)}\right\}}_{v=1}^{V}$ and ${\left\{Y_{p}\right\}}_{p=1}^{P}$;

2: Compute *L* by $L=\xi_{3}\left(\sum_{v=1}^{V}L^{\left(v\right)}+\delta \sum_{p=1}^{P}\beta_{p}L_{p}\right)$;

3: **repeat** 

4:  Update *F* by (16);

5:  Update *G* by (17);

6:  Update *R* by (18);

7: **until**  $\|R(t+1)-R(t){\|}_{2}<\epsilon$ or reach the maximum iterations $T_{2}$.

We use the same alternating optimization method for solving the objective function (15), whose optimization process is presented in the supplementary material, and the updating rules about *F*, *R*, and *G* are summed up as Algorithm 2 and can be shown as below


(16) $$F(i,j)\leftarrow F(i,j)\sqrt{\frac{\left(L^{\left(-\right)}F+{\left(\eta GR\right)}^{\left(+\right)}+\sigma F)(i,j\right)}{\left(L^{\left(+\right)}F+{\left(\eta GR\right)}^{\left(-\right)}+\eta F+\sigma FF^{T}F)(i,j\right)}},$$

(17) $$G_{i,j}=\{\begin{array}{c} 1\;\;\;j=\underset{k}{\arg\min}\|f^{\left(i\right)}-r_{k}{\|}_{F}^{2}\\ 0\;\;\;\text{else}, \end{array}$$

(18) R=UV,

where $r_{k}$  (k=1,…,c) is the column vectors of the matrix *R* and σ is an introduced parameter for constraint $F^{T}F=I$ in the supplementary material. *U* and *V* are left and right parts of the SVD decomposition of $G^{T}F$, of which proving process can be found in the literature (Huang et al. 2013). $L^{\left(+\right)}$ and $L^{\left(-\right)}$ are defined as


(19) $$L^{\left(+\right)}(i,j)=\frac{\left|L(i,j)|+L(i,j\right)}{2},$$

(20) $$L^{\left(-\right)}(i,j)=\frac{\left|L(i,j)|-L(i,j\right)}{2},$$

and so do ${\left(\eta GR\right)}^{\left(+\right)}$ and ${\left(\eta GR\right)}^{\left(-\right)}$.

Algorithm 3 seOMLR

**Input:** multi-view feature matrices ${\left\{X^{\left(v\right)}\right\}}_{v=1}^{V}$; base clustering matrices ${\left\{Y_{p}\right\}}_{p=1}^{P}$; parameters $\xi_{1}$, $\xi_{2}$, $\xi_{3}$, δ, η, and σ; maximum iterations *T*.

**Output:** Cluster indicator matrix *G*.

1: Initialize $E^{\left(v\right)}$, $Z^{\left(v\right)}$, $C^{\left(v\right)}$, $Q_{1}$, and $Q_{2}$ as zero matrices, ρ=1.2, $\epsilon =2\times 10^{-4}$;

2: Initialize *G* as a zero matrix,

3: Initialize *F* and *R* randomly;

4: **repeat** 

5:  Given *F*, compute $\beta_{p}$ by (7);

6:  Given *F*, obtain ${\left\{Z^{\left(v\right)}\right\}}_{v=1}^{V}$ and ${\left\{E^{\left(v\right)}\right\}}_{v=1}^{V}$ via Algorithm 1;

7:  Given ${\left\{Z^{\left(v\right)}\right\}}_{v=1}^{V}$, ${\left\{E^{\left(v\right)}\right\}}_{v=1}^{V}$, and ${\left\{\beta_{p}\right\}}_{p=1}^{P}$, obtain *F*, *R*, and *G* via Algorithm 2;

8: **until** convergence $\|{\left(GG^{T}\right)}_{t+1}-{\left(GG^{T}\right)}_{t}{\|}_{\infty }<0.05$ or reach the maximum iterations *T*.

Based on the aforementioned optimization process, we have summarized seOMLR in Algorithm 3.

## 3. Results

### 3.1. Experimental settings

#### 3.1.1. Parameter settings

In our model, these three free parameters $\xi_{1}$, $\xi_{2}$, and $\xi_{3}$ need to be set reasonably, and the performance of seOMLR can be enhanced by utilizing $\xi_{1}=\nu^{1-t}$, $\xi_{2}=\alpha$ and $\xi_{3}=\beta \nu^{t-1}$, where ν=1.2 and t∈{1,…,T} is the iteration index, inspired by Wang et al. (2017). Therefore, only two parameters α and β are required and vary within the range {0.0001, 0.001, 0.01, 0.1, 0,1, 10, 100, 1000}. The regularization parameter δ, which balances the weight between feature matrices and base clustering algorithms, is also in the same range with α and β. Parameters η and σ are chosen from the set {0.0001, 0.001, 0.01, 0.1, 1, 10, 100, 1000}, and σ is the Lagrange multiplier about the constraint $F^{T}F=I$. Although we can not provide a theoretical proof of convergence for seOMLR, our experiments demonstrate that seOMLR exhibits highly stable convergence in practice. Following ECMSC, we have empirically demonstrated the convergence of seOMLR for simulated data and TCGA data (Supplementary Figure S1-S2), and can observe that the proposed seOMLR method converges in 2∼6 iterations. The stopping criteria of seOMLR are set to $\|{\left(GG^{T}\right)}_{t+1}-{\left(GG^{T}\right)}_{t}{\|}_{\infty }<0.05$ which means that the cluster index remains unchanged, or the maximum number of iterations T=10 in Algorithm 3. With respect to the maximum iterations of Algorithm 1 and Algorithm 2, we refer to the parameters in ECMSC and spectral rotation for setting $T_{1}=30$ and $T_{2}=3000$ with which seOMLR can achieve stable clustering (Supplementary Figure S3).

#### 3.1.2. Compared methods

We compared seOMLR with related multi-omics clustering methods including iClusterPlus, SNF, iClusterBayes, NEMO, PartIES, MDICC, subtype-WESLR, k-means, moCluster, and spectral clustering (Ng et al. 2001) on simulated data and TCGA data. It is worth noting that we set the parameters based on the guidelines provided in the respective papers, aiming to select the best possible NMI or *P*-value for each method. The parametric settings for the competing methods used in our work are presented in the Supplementary Material.

### 3.2. Research on simulated data

Several computational experiments were conducted to evaluate the effectiveness of seOMLR using simulated datasets, and the process of generating these datasets is detailed in the supplementary material. The normalized mutual information namely NMI was used to evaluate the performance on simulated datasets. Three methods with favourable performance, namely SNF, iClusterPlus, and moCluster, are employed as inputs of seOMLR for ensemble learning to promote subspace learning. Experiments on simulated data indicate the robustness of seOMLR to various parameter settings (Supplementary Figure S4).

#### 3.2.1. Comparison across distinct extra noise

Datasets including 0%, 5%, and 15% extra noise, that is, low, moderate, and high noise, were randomly generated and repeated 50 times separately to guarantee the reliability of the experimental results. We compared the NMI values between the clusters obtained by different methods and the ground-truth clusters under distinct extra noise (Table 1; Supplementary Figure S5A). As described in Table 1, seOMLR demonstrates superior consistency with ground-truth clusters compared to other methods under various noise settings, exhibiting minimal fluctuation when introducing different levels of additional noise. MDICC and PartIES perform poorly, probably because the algorithms are sensitive to parameters. The moCluster, iClusterBayes, and iClusterPlus methods have also been relatively stable under different noise levels. SNF and subtype-WESLR have shown good performance across all noise levels, but are inferior to seOMLR.

**Table 1:** Performance of distinct methods on synthetic data.

Method	Low noise	Moderate noise	High noise
MDICC	0.05±0.01	0.02±0.01	0.02±0.02
PartIES	0.35±0.15	0.30±0.09	0.16±0.11
moCluster	0.37±0.04	0.40±0.03	0.40±0.11
iClusterBayes	0.48±0.15	0.51±0.12	0.50±0.13
iClusterPlus	0.50±0.06	0.50±0.07	0.50±0.07
NEMO	0.67±0.07	0.68±0.14	0.43±0.17
SNF	0.95±0.06	0.86±0.16	0.63±0.11
subtype-WESLR	0.91±0.02	0.87±0.17	0.71±0.10
seOMLR	0.98±0.04	0.93±0.12	0.79±0.15

Best results are in boldface.

#### 3.2.2. Better base clustering makes greater contributions for representation learning

**Figure 2:**
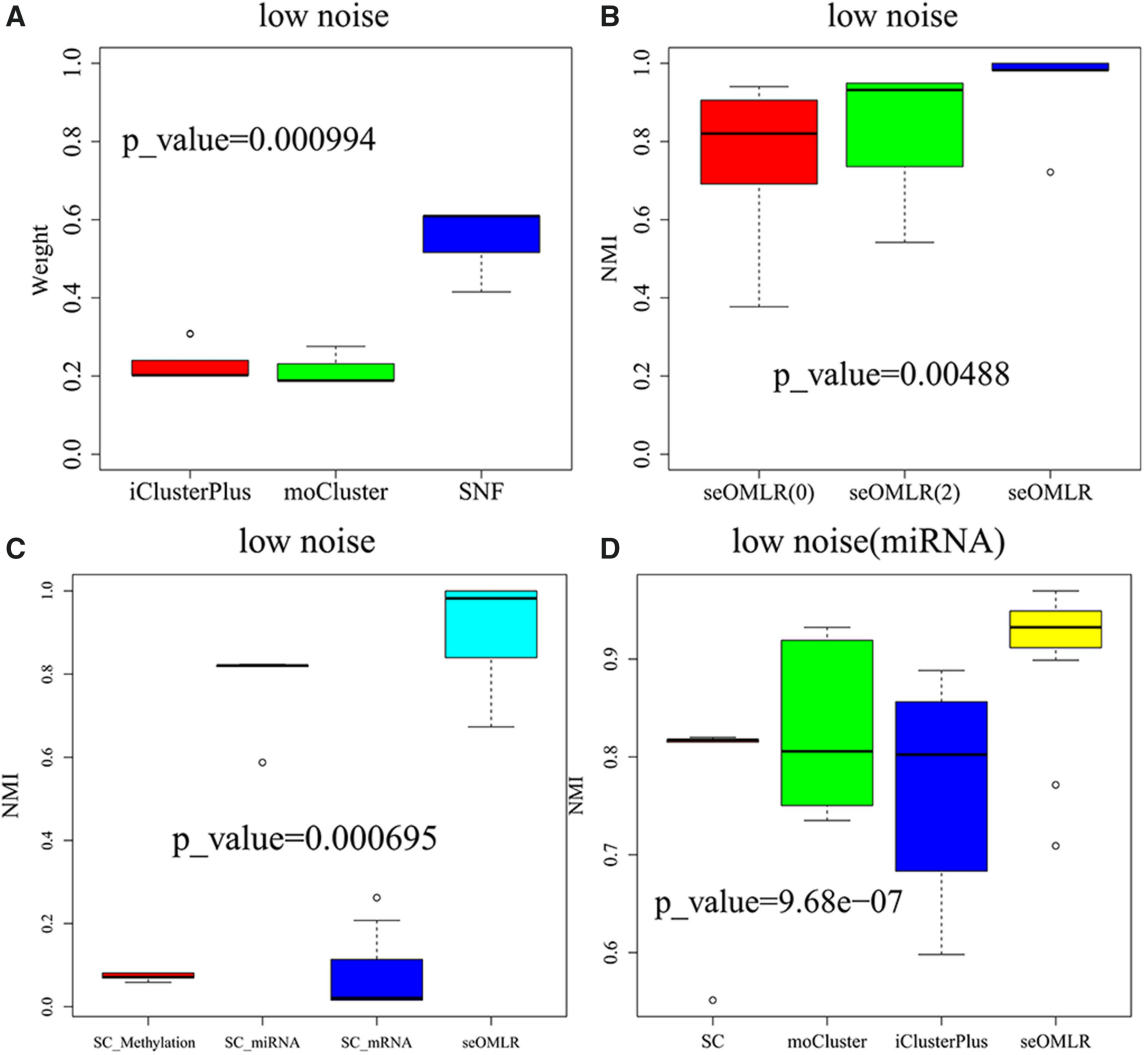
Analysis on synthetic data. Note that all p_values were obtained by two sample t-test. (A) Contribution of base methods to seOMLR. p_value =0.000994 indicates the significant difference between iClusterPlus and moCluster. (B) Values of NMI on seOMLR under distinct numbers of base clustering. p_value =0.00488 indicates the significant difference between seOMLR0 and seOMLR. (C) Values of NMI among SC-methylation, SC-miRNA, SC-mRNA and seOMLR using SC-methylation, SC-miRNA and SC-mRNA as base clustering. p_value =0.000695 indicates the significant difference between SC-miRNA and seOMLR. (D) Values of NMI among SC, moCluster, iClusterPlus, and seOMLR using the above methods as base methods on miRNA. p_value $=9.68\times 10^{-7}$ indicates the significant difference between iClusterPlus and seOMLR.

As a base method of seOMLR, SNF exhibits insensitivity to additional noise, with its clustering performance overall second only to seOMLR and subtype-WESLR which also adopts an ensemble learning strategy and employs the same base clustering methods as seOMLR. Furthermore, although moCluster and iClusterPlus performed poorly in identifying clusters, they were more stable under noise and were therefore selected as additional base methods. Performances of these three base methods correspond to contributions of base clustering to seOMLR, that is, better base clustering results lead to greater contributions to seOMLR in Fig. 2A, and SNF contributes the most.

#### 3.2.3. Effects of the number about basic clustering methods on seOMLR

We also discussed the validity of seOMLR while different numbers of base methods are regarded as inputs (Fig. 2B). SeOMLR0 refers to the absence of basic clusters as inputs, which can be achieved by setting δ=0. SeOMLR(2) adopts moCluster and iClusterPlus as its foundational methods due to their stable performance (Supplementary Figure S5B), while our complete model seOMLR additionally incorporates SNF alongside moCluster and iClusterPlus, as SNF demonstrates superior performance compared to both (Table 1). As described in Fig. 2B, seOMLR performs better than seOMLR0 and seOMLR(2), which indicates that well-performing base clustering results contribute to enhancing the performance of seOMLR. Moreover, when more high-performance basic clusters are used as inputs to seOMLR without parameter settings, the performance improvement is more significant to some extent, which is consistent with and superior to our previous work subtype-WESLR.

#### 3.2.4. Multi-omics data vs single-omic data

Spectral clustering is an efficient and straightforward method frequently employed in clustering research. We separately performed spectral clustering on DNA methylation, miRNA, and mRNA, and labelled them as SC-methylation, SC-miRNA, and SC-mRNA, whose clustering results were used as inputs of seOMLR. Fig. 2C demonstrates that integrating multi-omics data enables the utilization of some effective information from different data types, thereby facilitating the identification of common patterns more effectively than relying on single-omic data. As shown in Supplementary Figure S5C, we also discussed a scenario in which clustering results of SC-methylation, SC-miRNA, and SC-mRNA in any pairwise combinations were separately utilized as inputs to seOMLR. Experimental results show that seOMLR performs better on three data types compared to any combination of two data types, and indicate that integrating more multi-omics data of high quality can be more helpful for capturing common patterns. Since Fig. 2C demonstrates that miRNA performs better than DNA methylation and mRNA using spectral clustering on simulated data, we additionally conducted experiments on miRNA using spectral clustering named SC, moCluster, iClusterPlus, and seOMLR based on the aforementioned methods as basic approaches (Fig. 2D). SNF was not employed as a basic method because it does not work with a single data type. Results in Fig. 2D show that seOMLR holds true for single data type. In summary, various experimental results on simulated data demonstrate that seOMLR is more effective and robust than other state-of-the-art methods in identifying common patterns.

#### 3.2.5. One-step vs two-step

To demonstrate that the one-step strategy prevents information loss, we compared the full seOMLR with a variant where the algorithm stops at the continuous representation matrix F and uses k-means for discretization, referred to as seOMLR(two-step). Experimental results under varying levels of additional noise (Supplementary Figure S5D) show that the one-step strategy outperforms the two-step approach in the iterative optimization of seOMLR.

### 3.3. Research on TCGA data

The proposed seOMLR model was evaluated through several experiments to assess its performance and efficacy. There are nine existing approaches that are compared with seOMLR on publicly available multi-omics datasets from TCGA, arranged and provided by (Rappoport & Shamir 2018), including KIRC, BRCA, COAD, SKCM, LUSC, GBM, AML, and SARC (Supplementary Table S1). Each sample is comprised of the following data types: mRNA expression, miRNA expression, DNA methylation, and clinical profiles, of which preprocessing is displayed in the supplementary material. Supplementary Figure S6 illustrates how to select parameters for KIRC. Similar to the analysis on simulated data, we also examined the performance of seOMLR on TCGA KIRC data, comparing seOMLR with different omics combinations to single-omic. Specifically, we performed k-means clustering on three KIRC data types: k-means(miRNA), k-means(mRNA), and k-means(methy). These clustering results were then used as inputs for seOMLR in various combinations for ensemble learning. Survival analysis was conducted to compare the outcomes. Supplementary Figure S7 shows that integrating high-quality multi-omics data using seOMLR provides more effective cancer subtyping, consistent with the results observed in the simulated data.

#### 3.3.1. Comparison with competing methods on eight TCGA cancer cohorts

We compared seOMLR with nine competing methods of which moCluster, iClusterPlus, and SNF are basic methods as seOMLR on eight TCGA cancer data by the p_values (Table 2) and C_index values of the cox regression model. Notably, k-means was performed on integrated multi-omics data after concatenating DNA methylation, miRNA, and mRNA profiles. The negative $\mathrm{lo}g_{10}$  p_values in Table 2 indicate that seOMLR identifies cancer subtypes with greater precision than comparative methods, agreeing with the C_index values (Supplementary Table S2) of distinct methods in the most cases, which demonstrates seOMLR outperforms other approaches. Despite seOMLR yielding a slightly lower negative $\mathrm{lo}g_{10}$  p_value than subtype-WESLR on GBM, its C_index value remains the highest among all competing methods. Poor performance of MDICC on TCGA cancer data may be attributable to the algorithm’s sensitivity to parameter settings. To visually explore the differences among the identified subtypes, survival curves for eight cancers are displayed in Supplementary Figure S8-S9. For each method, we conducted enrichment analysis on six clinical labels including age, gender, pathological T, pathological M, pathological N, and pathological stage of eight cancer cohorts. As only age and gender data were available for GBM, AML, and SARC, enrichment analyses were performed solely on these two variables. Supplementary Figure S10 indicates that seOMLR exhibits stable performance across most cancer cohorts, with particularly favourable results on GBM and AML.

**Table 2:** Survival analysis of distinct methods on TCGA data.

Cancer type	KIRC	BRCA	COAD	SKCM	GBM	LUSC	AML	SARC
NEMO	4.48(3)	0.31(4)	0.96(4)	2.74(4)	2.96(3)	2.15(3)	1.31(6)	2.27(4)
iClusterPlus	1.92(2)	2.14(5)	1.04(4)	1.10(4)	0.82(3)	0.92(3)	3.04(5)	2.70(5)
iClusterBayes	2.51(4)	1.06(5)	0.89(4)	1.85(4)	0.22(3)	1.24(3)	1.77(4)	2.03(4)
moCluster	2.82(3)	3.31(5)	1.04(3)	2.96(4)	1.96(3)	2.31(3)	2.69(3)	2.18(5)
MDICC	0.45(3)	0.69(5)	1.56(5)	1.67(5)	0.47(5)	1.23(4)	1.31(4)	2.23(5)
SNF	3.40(3)	2.82(4)	1.07(3)	2.31(4)	2.92(3)	2.03(3)	1.37(6)	3.23(4)
PartIES	1.71(2)	5.33(4)	0.97(5)	1.28(3)	2.44(5)	1.75(4)	1.93(3)	2.79(4)
k-means	2.21(4)	2.54(5)	0.26(3)	1.03(3)	0.77(3)	0.50(3)	2.07(5)	2.21(5)
subtype-WESLR	4.76(4)	5.24(5)	2.43(4)	5.00(5)	3.84(3)	2.30(5)	2.99(4)	2.45(5)
seOMLR	5.09(5)	5.31(7)	3.43(8)	5.43(8)	3.07(6)	3.95(7)	5.38(6)	5.71(6)

Negative $\mathrm{lo}g_{10}$  p_value of Log-rank test is used for statistical signigicance test. Numbers of clusters are in parentheses. Best results are in boldface.

#### 3.3.2. Evaluation of subtypes identified in KIRC

Kaplan-Meier survival analysis was employed to analyse the subtypes identified by distinct methods on KIRC. As shown in Supplementary Figure S8, there are worst survival rates for subtype 1 (35 samples) obtained by seOMLR with a median survival time of 722 days, and best survival rates about subtype 5 (57 samples) in which over 86% samples are still alive at the end of the follow-up. Moreover, the significance of seOMLR ($-log_{10}$  p_value=5.09 and C_index=0.674) exceeds competing methods in Table 2 and Supplementary Table S2, respectively.

We further conducted differential expression analysis between any two KIRC subtypes identified by seOMLR using the R package edgeR (Robinson et al. 2010), aiming to discover differentially expressed mRNAs (*P*-adj value ≤0.05; FoldChange = 2) and miRNAs (*P*-adj value ≤0.05; FoldChange = 1.5). These differentially expressed mRNAs from two combinations of all subtypes are presented in Supplementary Figure S11 as heatmaps, with the merged data stored in KIRC-differential-mRNA.csv. It can be seen that the differentially expressed mRNAs identified can provide intuitive differentiation for any two subtypes as a whole, indicating that the identified subtypes have substantial significance and interpretability.

For resolving the biological functional associations and synergistic interactions among all identified differentially expressed mRNAs, Gene Ontology (GO) terms and KEGG pathway enrichment analyses were performed on all differentially expressed mRNAs in KIRC using the DAVID (Sherman et al. 2022) tool. Supplementary Figure S12 displays the differentially expressed mRNAs enriched in GO semantic terms and KEGG pathways, which are implicated in the malignant tumourigenesis, metabolism, invasion, and prognosis.

We also explored the signalling pathways potentially involved in differentially expressed miRNAs using predicted targets from experimentally validated miRNA interactions within the DIANA-TarBase database, leveraging the DIANA-miRPath (Spyros et al. 2023) tool. These differentially expressed miRNAs participate in several pathways associated with tumourigenesis, progression, and metastasis (Supplementary Figure S13). It has also been demonstrated that microRNAs such as miR-378c, miR-429, miR-1299, and miR-3941 play a significant role in promoting kidney tumor initiation, growth, and metastasis (Lin & Cai 2020, Ma et al. 2020, Yu et al. 2020).

#### 3.3.3. Evaluation of subtypes identified in GBM

**Figure 3:**
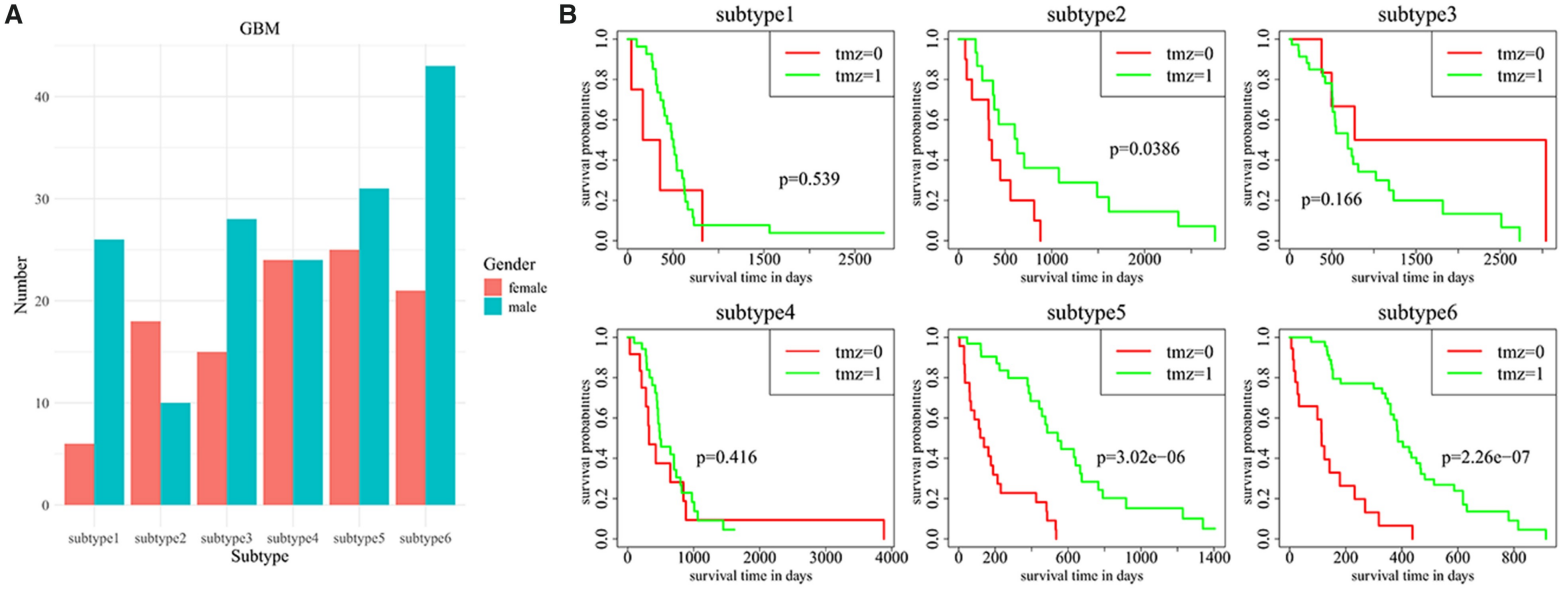
Analysis of identified subtypes on GBM by seOMLR. (A) The bar chart of gender across six subtypes. (B) Survival analysis of patients treated with TMZ in six subtypes. TMZ =0 indicates patients not treated by TMZ and TMZ =1 indicates patients treated by TMZ.

The six subtypes were identified on GBM via seOMLR, which can be associated with the previously reported subtypes based on molecular typing and molecular characteristics (Supplementary Table S3; Fig. 3). Generally speaking, GBM-related subtypes are divided into classical, mesenchymal, neural, and proneural subtypes based on mRNA data, which also can be classified into G-CIMP and non-G-CIMP subtypes according to differences in the CpG island methylation phenotype (CIMP) derived from DNA methylation data. We also studied the gender distribution and survival analysis of patients treated with temozolomide (TMZ) and those without TMZ treatment for each subtype. Supplementary Table S3 shows the distribution of the number of samples based on mRNA and methylation data in the identified six clusters. It can be seen that subtypes 1 corresponding to the Mesenchymal subtype and non-G-CIMP subtype are mostly male, and subtype 3 can be classified as the proneural subtype in which G-CIMP and non-G-CIMP are comparable in number. Subtype 2, mostly female, does not correspond well to the reported mRNA-based subtypes, possibly due to its small sample size, but exhibits sensitivity to TMZ treatment, similar to subtypes 5 and 6. Subtype 4 may belong to the classical subtype with a balanced male-to-female ratio, demonstrating insensitivity to TMZ treatment.

#### 3.3.4. Evaluation of subtypes identified in other tumors

To validate the subtyping results from seOMLR, we compared the obtained subtypes with those previously reported for BRCA based on molecular typing and characteristics of PAM50 RNAseq (Supplementary Tables S4-S5). We also analysed the age distribution of the seven subtypes, as shown in Supplementary Figure S14. Detailed elaborations are provided in the supplementary material. Additionally, we performed differential expression analysis using mRNA data for BRCA, AML, and COAD to assess the biological relevance of the identified subtypes. Supplementary Figure S16 presents heatmaps of differentially expressed mRNA among the six AML subtypes identified by seOMLR, clearly illustrating the gene expression differences across subtypes. Furthermore, GO term and KEGG pathway enrichment analyses were conducted on differentially expressed mRNAs from BRCA, AML, and COAD (Supplementary Figures S15, S17, S19), with results provided in the supplementary material. We also explored signaling pathways potentially involved in differentially expressed miRNAs in AML and COAD using predicted targets from experimentally validated miRNA interactions via the DIANA-miRPath tool (Supplementary Figures S18, S20). These analyses are also detailed in the supplementary material. Experiments across various datasets indicate the applicability of seOMLR and the biological significance of the identified subtypes.

## 4. Conclusion

Cancer is a complex disease characterized by significant molecular heterogeneity and diverse clinical manifestations, motivating the pursuit of precise subtyping through multi-omics data. In this paper, a novel multi-view latent representation model named seOMLR was proposed, which learns the specificity and consistency of multi-omics data and integrates fusion and clustering into a unified optimization framework to identify cancer subtypes. SeOMLR can fully exploit the consistency and specificity of multi-omics data through relaxed exclusivity constraints and consistency regularization terms, and provide in-depth insight into the valuable pattern information of other methods using the self-weighted ensemble strategy to indirectly enhance consistency and specificity learning. Besides, spectral rotation was introduced to extract clustering structures, and enabled mutual reinforcement between clustering and fusion. To justify the applicability and effectiveness of seOMLR, we conducted experiments on simulated datasets and eight publicly multi-omics datasets from TCGA. Experimental results on both simulated and TCGA datasets demonstrate that seOMLR outperforms competing methods in most cases. It fully exploits the consistency and specificity of heterogeneous data during training, and its integration strategy combined with self-weighted ensemble learning effectively improves model robustness while uncovering more reliable patterns for cancer subtyping research. However, seOMLR currently relies on PCA for dimensionality reduction during preprocessing. Although this improves computational tractability, it mainly captures linear structure and makes the link between inferred subtypes and individual molecular features indirect. Accordingly, biological interpretation at the feature level is conducted post-hoc in the original feature space, and intrinsic feature attribution is beyond the current scope. Future work will explore integrating deep autoencoders to model non-linear relationships and extending seOMLR to better connect subtype assignments with molecular features. Additionally, within seOMLR, we did not address the relative contribution of each data type to subtype identification, which is a limitation of our current approach and represents a potential direction for future improvement.

## Supplementary Material

btag074_Supplementary_Data

## Data Availability

The data used in simulation study was generated by subtype-WESLR. TCGA-KIRC, TCGA-BRCA, TCGA-COAD, TCGA-SKCM, TCGA-GBM, TCGA-LUSC, TCGA-AML, and TCGA-SARC are publicly available at https://portal.gdc.cancer.gov/, and we acquired from http://acgt.cs.tau.ac.il/multi_omic_benchmark/download.html. Source codes of seOMLR are available at https://github.com/songwenjing123/seOMLR.
