# Supplementary material for: SeOMLR: one-step multi-view latent representation with self-weighted ensemble learning for multi-omics cancer subtyping
Source: Bioinformatics. 2026 Mar 5;42(3):btag074. doi: 10.1093/bioinformatics/btag074 (PMC12980331; doi:10.1093/bioinformatics/btag074)
Supplement: btag074_Supplementary_Data [file btag074_supplementary_data.zip › supplementary material.pdf]

Supplementary material of seOMLR: one-step multi-view latent representation with self-weighted ensemble learning for multi-omics cancer subtyping

Wenjing Song, School of Science, Southwest Petroleum University, Chengdu, 610500, China.
Yesen Sun, School of Arts and Sciences, Guangzhou Maritime University, Guangzhou, 510725, China.
Le Ou-Yang, Faculty of Engineering, Shenzhen MSU-BIT University, Shenzhen, 518172, China.
Email: sunyesen@gzmtu.edu.cn; leouyang@smbu.edu.cn.

Methods

Self-weighted ensemble learning

Learning the subtyping information of different clustering methods with an ensemble strategy can effectively promote cluster accuracy for identifying cancer subtypes, which has been demonstrated in our previous work subtype-WESLR [1] where it is necessary to adjust parameters to achieve ensemble learning. Here, a self-weighted ensemble learning strategy will be designed without introducing additional parameters in seOMLR.

The effective information of basic clustering can be reflected by the graph Laplacian matrix, which is applied to ensemble learning to adaptively optimize the subspace F. Base indicator consistency of distinct clustering for F can be obtained by

$$\begin{aligned} &\arg\min_{F} Tr\left(F^T\left(\sum_{p=1}^{P}\beta_p L_p\right)F\right) \\ &s.t. \quad F^T F = I, F>0, \end{aligned} \tag{1}$$

where the weight coefficient β_p balances the contribution of the p-th base clustering method to the prediction.

Similar to AMGL [2], consider the following form

$$\begin{aligned} &\arg\min_{F} \sqrt{Tr\left(F^{T}\left(\sum_{p=1}^{P} L_{p}\right)F\right)} \\ &s.t. \quad F^{T}F = I, F{>}0. \end{aligned} \tag{2}$$

The Lagrange function of problem (2) about F can be written as

$$\sqrt{Tr\left(F^{T}\left(\sum_{p=1}^{P} L_{p}\right)F\right)} + \Psi(\Lambda, F), \tag{3}$$

where Λ is the Lagrange multiplier and $\Psi(\Lambda, F)$ is the formalized term composed of the constraints about F.

Let the derivation of (3) on F be 0, we have

$$\sum_{p=1}^{P} \beta_{p} \frac{\partial Tr\left(F^{T} L_{p} F\right)}{\partial F} + \frac{\partial \Psi(\Lambda, F)}{\partial F} = 0, \tag{4}$$

where

$$\beta_{p} = \frac{1}{2\sqrt{Tr\left(F^{T} L_{p} F\right)}} \qquad (p = 1, \ldots, P) \tag{5}$$

is dependent on the subspace F. Note that (4) can not be directly solved, but when β_p is set to be stationary, (4) can be considered as the solution to (1) which looks easier to be solved. Therefore, we can take an alternating optimization strategy to compute F and β_p iteratively by (1) and (5), respectively.

Supposing that F can be calculated by (1) while β_p is fixed, this F will be continuously used to update β_p according to (5), which inspires us to take an alternating optimization strategy to compute F and β_p iteratively.

Optimization

Suppose $L = \xi_3 \left(\sum_{v=1}^{V} L^{(v)} + \delta \sum_{p=1}^{P} \beta_p L_p \right)$, the objective function of seOMLR for cancer subtyping can be reformulated as

$$\begin{aligned} \min_{\substack{\{E^{(v)}\}_{v=1}^{V},F,\\ \{Z^{(v)}\}_{v=1}^{V},G,\\ \{\beta_p\}_{p=1}^{P},R}} \sum_{v=1}^{V} & \left(\left\|E^{(v)}\right\|_1 + \xi_1 \left\|Z^{(v)}\right\|_1 + \xi_2 \sum_{\substack{w=1\\ w\neq v}}^{V} \left\|Z^{(v)} \odot Z^{(w)}\right\|_1 \right) \\ & + Tr\left(F^T L F\right) + \eta \left\|F - GR\right\|_F, \\ & s.t. \\ & X^{(v)} = X^{(v)} Z^{(v)} + E^{(v)}, \\ & diag\left(Z^{(v)}\right) = 0,\ v = 1, \ldots, V, \\ & F^T F = I, F>0,\ R^T R = I, \\ & G \in Ind,\ G \in \{0,1\}^{n\times c}, \end{aligned} \tag{6}$$

where $Z^{(v)} \in R^{n\times n}$ and $E^{(v)} \in R^{n\times n}$ are the self-representation matrix and error matrix of the v-th view, respectively. Weight coefficient β_p balances the contribution of the p-th base clustering method to the prediction. I is an identity matrix, and $F^T F = I$ and $R^T R = I$ mean the normalized orthonormal constraint imposed on the F and R. $G \in Ind$ denotes G is an indicator matrix of which the unique 1 in each row vector indicates its cluster membership. $\xi_1>00$, $\xi_2>0$, $\xi_3>0$, and $\eta>0$ are regularization parameters. $\delta \geq 0$ is used to balance feature matrices and base ensemble clustering results.

For the v-th ($v = 1, \ldots, V$) view, we initialize $E^{(v)}$ and $Z^{(v)}$as zero matrices. The initial elements in matrices F and R are uniformly distributed random numbers between 0 and 1. The initial G is an indicator matrix whose elements are all zeros. Besides, we set the initial values of β_p ($p = 1, \ldots, P$) to $1/P$ to ensure that all base ensemble clustering results have equal importance, and optimize the objective function (9) by alternately and iteratively updating to obtain the solutions, as analyzed in the supplementary material, in which we optimize the value of β_p, $E^{(v)}$, and $Z^{(v)}$ given F; and then employ them to update F, R, and G. The details are in the following.

Step 1: update β_p, $E^{(v)}$, and $Z^{(v)}$

When F, R, and G and are fixed, we update β_p by (5), and the objective function (6) is written with respect to $E^{(v)}$ and $Z^{(v)}$ as

$$\begin{aligned}&\underset{\{E^{(v)},Z^{(v)}\}_{v=1}^{V}}{\arg\min}\sum_{v=1}^{V}\left(\|E^{(v)}\|_1+\xi_1\|Z^{(v)}\|_1+\xi_2\sum_{\substack{w=1\\w\neq v}}^{V}\|Z^{(v)}\odot Z^{(w)}\|_1+\xi_3 Tr\left(F^TL^{(v)}F\right)\right)\\&\quad s.t.\\&\quad X^{(v)}=X^{(v)}Z^{(v)}+E^{(v)},\\&\quad diag\left(Z^{(v)}\right)=0,\ v=1,\ldots,V.\end{aligned} \tag{7}$$

We solve for $E^{(v)}$ and $Z^{(v)}$ separately on each v ($v=1,\ldots,V$) by optimizing the following sub-problem

$$\begin{aligned}&\underset{E^{(v)},Z^{(v)}}{\arg\min}\|E^{(v)}\|_1+\xi_1\|Z^{(v)}\|_1+\xi_2\sum_{\substack{w=1\\w\neq v}}^{V}\|Z^{(v)}\odot Z^{(w)}\|_1+\xi_3 Tr\left(F^TL^{(v)}F\right)\\&\quad s.t.\\&\quad X^{(v)}=X^{(v)}Z^{(v)}+E^{(v)},\\&\quad diag\left(Z^{(v)}\right)=0.\end{aligned} \tag{8}$$

The above problem (8) is equivalent to

$$\begin{aligned}&\underset{E^{(v)},Z^{(v)}}{\arg\min}\|E^{(v)}\|_1+\xi_1\|Z^{(v)}\|_1+\xi_2\sum_{\substack{w=1\\w\neq v}}^{V}\|Z^{(v)}\odot Z^{(w)}\|_1+\xi_3 Tr\left(F^TL^{(v)}F\right)\\&\quad s.t.\\&\quad X^{(v)}=X^{(v)}C^{(v)}+E^{(v)},\\&\quad C^{(v)}=Z^{(v)}-diag\left(Z^{(v)}\right).\end{aligned} \tag{9}$$

We solve this problem using the Alternating Direction Method of Multipliers (ADMM) [3], and the augmented Lagrangian can be expressed as

$$\begin{aligned}l(Z^{(v)},C^{(v)},E^{(v)},Q_1,Q_2)&=\xi_1\|Z^{(v)}\|_1+\xi_2\sum_{\substack{w=1\\w\neq v}}^{V}\|Z^{(v)}\odot Z^{(w)}\|_1+\xi_3\mathrm{Tr}\left(F^TL^{(v)}F\right)\\&+\|E^{(v)}\|_1+\Phi\left(Q_1,X^{(v)}-X^{(v)}C^{(v)}-E^{(v)}\right)+\Phi\left(Q_2,C^{(v)}-Z^{(v)}+diag\left(Z^{(v)}\right)\right),\end{aligned} \tag{10}$$

where Q_1 and Q_2 are Lagrange multipliers, and $\Phi(Q,Y)=\frac{\mu}{2}\|Y\|_F^2+\langle \mathrm{Q,Y}\rangle$, of which $\langle\cdot,\cdot\rangle$ indicates the matrix inner product and μ is a positive penalty scalar.

To resolve the aforementioned problem (10), we employ an iterative approach to update $Z^{(v)}$, $C^{(v)}$, $E^{(v)}$, Q_1, and Q_2. Furthermore, note that the objective

$Tr\left(F^T L^{(v)} F\right)$ can be rewritten as:

$$Tr\left(F^T L^{(v)} F\right) = \sum_{i,j=1}^{n} |z_{i,j}| \left(\frac{1}{2}\|f^i - f^j\|_F^2\right) = \|Z^{(v)} \odot D\|_1, \tag{11}$$

where D is a $n \times n$ matrix with elements $D_{i,j} = \sum_{i,j=1}^{n} \frac{1}{2}\|f^i - f^j\|_F^2$, of which f^i is the i-th row vector of F. Consequently, When $C^{(v)}$, $E^{(v)}$, Q_1, and Q_2 are fixed, the Lagrangian function (10) about $Z^{(v)}$ becomes

$$\xi_1\|Z^{(v)}\|_1 + \xi_2 \sum_{\substack{w=1\\w\neq v}}^{V} \|Z^{(v)} \odot Z^{(w)}\|_1 + \xi_3\|Z^{(v)} \odot D\|_1 + \Phi\left(Q_2, C^{(v)} - Z^{(v)} + diag\left(Z^{(v)}\right)\right). \tag{12}$$

We can further arrange (12) to obtain

$$\left\|Z^{(v)} \odot \left(\xi_1 E + \xi_2 \sum_{\substack{w=1\\w\neq v}}^{V} \|Z^{(w)}\|_1 + \xi_3\|D\|_1\right)\right\|_1 + \frac{\mu}{2}\left\|C^{(v)} + \frac{Q_2}{\mu} - \left(Z^{(v)} - diag\left(Z^{(v)}\right)\right)\right\|_F^2, \tag{13}$$

where $E \in R^{n\times n}$ is a matrix with all elements equal to 1. In accordance with [4], the closed-form solution for $Z^{(v)}$ can be computed by

$$\begin{aligned} \hat{Z}^{(v)} &= \mathcal{S}_{\frac{1}{\mu}(\xi_1 E + \xi_2 \sum_{w=1,w\neq v}^{V} Z^{(w)} + \xi_3 D)}\left[C^{(v)} + \frac{Q_2}{\mu}\right], \\ Z^{(v)} &= \hat{Z}^{(v)} - diag(\hat{Z}^{(v)}), \end{aligned} \tag{14}$$

in which $\mathcal{S}_\tau[\cdot]$ is the shrinkage thresholding operator.

Similarly, while $Z^{(v)}$, $E^{(v)}$, Q_1, and Q_2 are fixed, the Lagrangian function (10) is written with respect to $C^{(v)}$ as

$$\Phi(Q_1, X^{(v)} - X^{(v)}C^{(v)} - E^{(v)}) + \Phi(Q_2, C^{(v)} - Z^{(v)} + diag(Z^{(v)})). \tag{15}$$

By differentiating the function (15) about $C^{(v)}$ and making it to be 0, we have

$$\begin{aligned} C^{(v)} = \left(X^{(v)T} X^{(v)} + I\right)^{-1} &\left[X^{(v)T}\left(X^{(v)} - E^{(v)} + \frac{Q_1}{\mu}\right)\right. \\ &\left. + Z^{(v)} - diag\left(Z^{(v)}\right) - \frac{Q_2}{\mu}\right]. \end{aligned} \tag{16}$$

Fixing $Z^{(v)}$, $C^{(v)}$, Q_1, and Q_2, the associated optimization problem (10) respecting $E^{(v)}$ turns into

$$\|E^{(v)}\|_1 + \Phi(Q_1, X^{(v)} - X^{(v)}C^{(v)} - E^{(v)}), \tag{17}$$

of which solution can be computed with

$$E^{(v)} = \mathcal{S}_{\frac{1}{\mu}}\left[X^{(v)} - X^{(v)}C^{(v)} + \frac{Q_1}{\mu}\right]. \tag{18}$$

Without loss of generality, we can update Q_1 and Q_2 by

$$\begin{aligned} Q_1 &= Q_1 + \mu\left(X^{(v)} - X^{(v)}C^{(v)} - E^{(v)}\right), \\ Q_2 &= Q_2 + \mu\left(C - Z^{(v)} + diag(Z^{(v)})\right), \end{aligned} \tag{19}$$

when $Z^{(v)}$, $C^{(v)}$, and $E^{(v)}$ are fixed.

Step 2: update F, R, and G

Fixed $Z^{(v)}$, $E^{(v)}$, and β_p, (6) becomes as in relation to F, R, and G, i.e.,

$$\begin{aligned} &\underset{F,G,R}{\arg\min} Tr\left(F^T LF\right) + \eta\|F - GR\|_F, \\ &\quad s.t. \\ &\quad F^T F = I, F>0,\ R^T R = I, \\ &\quad G \in Ind,\ G \in \{0,1\}^{n\times c} \end{aligned} \tag{20}$$

While R and G are fixed, the function (20) about F is written as

$$\begin{aligned} &\underset{F,G,R}{\arg\min} Tr\left(F^T LF\right) + \eta\|F - GR\|_F, \\ &\quad s.t. \\ &\quad F^T F = I, F>0. \end{aligned} \tag{21}$$

The Lagrangian function of (21) about F changes into

$$l(F,\sigma,\phi) = Tr(F^T LF) + \eta\|F - GR\|_F + \frac{\sigma}{2}\|F^T F - I\|_F^2 - Tr(\phi F), \tag{22}$$

where σ and ϕ are Lagrangian multipliers. Let the derivation of (22) on F be 0, we have

$$\phi = (L + \eta I)F - \eta GR - \sigma F + \sigma F F^T F. \tag{23}$$

Besides, we can obtain $\phi(i,j)F(i,j) = 0$ by applying the KKT condition. We get

$$F(i,j) \leftarrow F(i,j)\sqrt{\frac{(L^{(-)}F + (\eta GR)^{(+)} + \sigma F)(i,j)}{(L^{(+)}F + (\eta GR)^{(-)} + \eta F + \sigma F F^T F)(i,j)}}, \tag{24}$$

where

$$L^{(+)}(i,j) = \frac{|L(i,j)| + L(i,j)}{2}, \tag{25}$$

$$L^{(-)}(i,j) = \frac{|L(i,j)| - L(i,j)}{2}. \tag{26}$$

So do $(\eta GR)^{(+)}$ and $(\eta GR)^{(-)}$.

When F is fixed, the function (20) in relation to R and G becomes

$$\begin{aligned} &\arg\min_{G,R} \eta \|F - GR\|_F, \\ s.t. \quad &R^T R = I, G \in Ind,\ G \in \{0,1\}^{n \times c} \end{aligned} \tag{27}$$

As described in [5], we fix R in (27), and update G by

$$G_{i,j} = \begin{cases} 1 & j = \arg\min_k \|f^{(i)} - r_k\|_F^2, \\ 0 & \text{else}, \end{cases} \tag{28}$$

where $r_k (k = 1, \ldots, c)$ is the column vectors of matrices R.

When G is fixed in (27), renew R by

$$R = UV, \tag{29}$$

where U and V are left and right parts of the SVD decomposition of $G^T F$, of which proving process can be found in the paper [5].

Results

Parametric settings on competing methods

Worthy of note is that we set parameters according to the rules in its paper, and conducted many tests with different settings on our simulated data or real data, attempting to choose the relatively better NMI or p_value for each method. The results in our work may be different from their reports due to difference in data and parameter settings.

PartIES: The hyper-parameters in PartIES are primarily related to the number of neighbors k and σ for kernel calculation. As PartIES is not very sensitive to the choices of k, we set $k = 20$ on all datasets in our work. σ varies within the range $\{0.2, 0.4, 0.6, 0.8, 1,1.2, 2, 10, 100\}$. Other parameters shall use their default values.

SNF: The method is implemented by the R package "SNFtool". The hyper-parameters of SNF contain μ in the range of [0.3, 0.8] and the number of neighbors K. As SNF is not very sensitive to K, we set $K = 20$ on all datasets in our work.

moCluster: The method is implemented in the Bioconductor package "mogsa". The performance of moCluster is mainly dependent on the number of joint latent variable, which is in the range of [2, 20] on all datasets in our work. Other parameters shall use their default values.

NEMO: The robustness of NEMO is related to the number of neighbors k, which is chosen from the set {25,35,45,...,105} in our work.

iClusterPlus: The method is implemented in the Bioconductor R package "iClusterPlus". The performance of iClusterPlus primarily depends on the number of eigen features which varies within the range [1, 7], and the vector of elastic net penalty terms α, whose elements are set to 1. All data types are set as "gaussian". Other parameters shall use their default values.

iClusterBayes: The method is implemented in the Bioconductor R package "iClusterPlus". The performance of iClusterBayes is mainly dependent on the number of eigen features, which ranges from 1 to 7, and the prior probability of the indicator variable γ for each dataset, which is set at 0.3. All data types are set as "gaussian". Other parameters shall use their default values.

MDICC: The parameters used in MDICC contain the number of k-neighbors k_1 when calculating the affinity matrix, the number of nearest neighbors of hyperparameters γ, and the first C eigenvectors of fusion network S with the largest eigenvalue. As MDICC is insensitive to k_1, we use its default value $k_1 = 18$. k_2 varies within the default range [41, 44], and C also uses its default value in our work.

subtype-WESLR: The hyper-parameters involved in subtype-WESLR mainly contain μ, λ, δ, and σ, of which values are in the range $\{0.0001,0.001,0.01,0.1,1,10, 100,1000,10000\}$. Other parameters shall use their default values.

k-means: Due to the instability of the clusters produced by the k-means algorithm, we repeated the process ten times and selected the optimal outcome for each dataset.

spectral clustering: We employ the same method as SNF to compute the similarity matrix for each data type.

Data simulation

We compared seOMLR with other methods based on the synthetic datasets involving miRNA, mRNA, and DNA methylation. Multi-view data were separately produced from real genomic profiles GSE73002[6], GSE10645[7], and GSE51557[8] for miRNA expression, mRNA expression, and DNA methylation data, as thoroughly explained by singular value decomposition(SVD) in the supporting information of Shi et al.[9].

To preserve all the biological characteristics of data, we only modified the right singular matrix. The original left singular matrix, diagonal matrix, and the modified right singular matrix which contains incomplete information of clusters, generated new matrix for each data type. The new matrix preserves the biological characteristics of each data type, and cannot distinguish the exact clusters. Integrating three incomplete information of clusters can discriminate exact clusters, i.e., common patterns. Therefore, three synthetic data have biological characteristics of each data type and common patterns among three data types.

Owing to the better performance of good-condition numeric examples over bad-condition [9], synthetic data were simulated in bad-condition with $mean^s \in \{0, 0.25, 0.5, 0.75\}$, including 200 samples with four ground-truth clusters as 1-50, 51-100, 101-150, and 151-200. Each data type can discriminate incomplete clusters, and all of the data types correspond to clusters {1-50, 51-150, 151-200}, {1-50/101-150, 51-100, 151-200}, and {1-100, 101-150, 151-200}. Specifically, the process is as follows.

From each real dataset, 200 samples were randomly selected from three datasets and referred to as X_1, X_2, and X_3, where rows and columns present features and samples, respectively. Then, singular value decomposition is separately performed on data matrices X_1, X_2, and X_3, i.e.,

$$X_1 = U_1 D_1 V_1, X_2 = U_2 D_2 V_2, X_3 = U_3 D_3 V_3. \tag{30}$$

To maintain the biological characteristics of data, we only modified the matrix V [9] for four pre-defined cluster structures. Hence, we separately constructed three novel data matrices X_{sim1}, X_{sim2}, and X_{sim3} of the same dimension as X_1, X_2, and X_3 with element

$$X_{ij} = mean^s + \epsilon_{ij}, \tag{31}$$

where $\epsilon_{ij} \sim N(0, 1)$ represents random bias for expression of element i in sample j and $mean^s \in \{0, 0.25, 0.5, 0.75\}$ represents the average expression level in the corresponding cluster.

We calculated the SVD of X_{sim1}, X_{sim2}, and X_{sim3} to create substitutional orthogonal matrices for matrix V (V_1, V_2, and V_3), with explicit cluster struc-

ture, i.e.,

$$X_{sim1} = U_{sim1}D_{sim1}V_{sim1}, X_{sim2} = U_{sim2}D_{sim2}V_{sim2}, X_{sim3} = U_{sim3}D_{sim3}V_{sim3}. \tag{32}$$

Finally, the synthetic datasets X_{new1}, X_{new2}, and X_{new3} were generated by

$$X_{new1} = U_1D_1V_{sim1}, X_{new2} = U_2D_2V_{sim2}, X_{new3} = U_3D_3V_{sim3}. \tag{33}$$

Preprocessing on TCGA data

To enhance the quality of experimental results, we performed preprocessing and standardization on TCGA data as described in [1], resulting in obtaining 206 samples in KIRC, 623 in BRCA, 214 in COAD, 439 in SKCM, 271 in GBM, 337 in LUSC, 159 in AML, and 261 in SARC shown in Table S1. Additionally, we performed Principal Component Analysis (PCA) for dimensionality reduction on each data type separately, retaining 95% information to eliminate data redundancy or noise. Determining the number of cancer subtypes is a critically important issue, as there is currently no unified and effective method available. In order to ascertain the most appropriate number of clusters, there are assumptions made that the number ranges from two to eight clusters, with the optimal number being that yielding the best p_value. Meanwhile, we permit competing methods to yield different optimal cluster numbers and disregard identified subtypes with clusters of size below nine across each methods.

Evaluation of subtypes identified in other tumors.

Evaluation of subtypes identified in BRCA. To validate the validity of seOMLR's subtyping results, we compared the obtained subtypes with those previously reported in BRCA based on molecular typing and molecular characteristics (Supplementary Tables S4-S5). Based on PAM50 RNA sequencing, BRCA-associated subtypes can be categorized as luminal-A, luminal-B, HER2-enriched, basal-like, and normal-like. The luminal-A and luminal-B subtypes are positive for oestrogen receptor (ER) and progesterone receptor (PR), but negative for human epidermal growth factor receptor 2 (HER-2). The HER2-enriched subtype is characterized by HER-2 positivity alongside ER and PR negativity. The basal-like subtype exhibits triple negativity for ER, PR, and HER-2. As illustrated in Supplementary Table S4-S5, subtype 1 corresponds to Basal-like. Subtype 3 and subtype 5 are associated with luminal-B and luminal-A. Subtype 7 also can be classified to luminal-A. HER2-enriched and normal-like cannot be corresponded well to the identified subtypes, which may be due to the small number of samples. We also studied the age distribution of seven subtypes in Supplementary Figure S14. Subtype 1 had the youngest average age at diagnosis, lower than that of subtype 4, which had the highest diagnosis age, with significant difference ($p-$value $= 0.026$) by two sample t-test.

Furthermore, we performed differential expression analysis with mRNA data on BRCA to explore whether the identified subtypes possess biological significance. GO terms and KEGG pathway enrichment analysis were separately

conducted on differentially expressed mRNAs of BRCA. As shown in Supplementary Figure S15A, cancer-related GO semantic terms about BRCA contain key functional modules such as signal transduction, positive regulation of transcription by RNA polymerase II, positive regulation of cell population proliferation, negative regulation of apoptotic process, plasma membrane, chromatin, cytoplasm, extracellular exosome, DNA-binding transcription factor activity, protein kinase activity, signaling receptor binding and so on, which play pivotal roles in malignant tumours initiation, progression, metastasis, and therapy. For example, signal transduction drives the activation of key pathways such as PI3K-AKt, forming the direct basis for targeted therapies, and the oestrogen receptor activates oncogenes such as MYC and CCND1 through positive regulation of transcription by RNA polymerase II pathways, thereby determining the molecular subtype and endocrine therapy sensitivity of hormone receptor-positive breast cancer. Differentiated BRCA mRNA expressions are mainly focused within KEGG cancer-related pathways, including cGMP-PKG signaling pathway, PI3K-Akt signaling pathway, MAPK signaling pathway, focal adhesion and so on (Supplementary Figure S15B). These pathways are closely associated with the initiation, progression and metastasis of cancer, offering insights for cancer treatment. For instance, the PI3K-Akt signalling pathway and MAPK signalling pathway constitute the treatment-driving pathways in breast cancer. Both directly correspond to approved therapeutic agents, forming the dual core of targeted therapy.

In a word, the identified subtypes on BRCA are reasonable and have statistical interpretation and biological significance.

Evaluation of subtypes identified in AML. Differential expression analysis were separately performed with mRNA and miRNA data on AML subtypes identified by seOMLR.

Supplementary Figure S16 presents the heatmaps of differentially expressed mRNA among six AML subtypes identified by seOMLR, which intuitively displays the differences of genes across different cancer subtypes, and indirectly demonstrates the biological significance of the identified subtypes.

We also conducted GO terms and KEGG pathway enrichment analysis on differentially expressed mRNAs of AML (Supplementary Figure S17). Supplementary Figure S17A indicates that cancer-related GO semantic terms of AML encompass key functional modules such as signal transduction, cell adhesion, cell differentiation, cell population proliferation, positive regulation of gene expression, plasma membrane, extracellular region, signaling receptor activity, growth factor activity, integrin binding and so on, which play pivotal roles in AML initiation, progression, metastasis, and therapy. For example, the core feature of AML is impaired differentiation of myeloid progenitor cells, leading to abnormal proliferation of immature cells, which is closely associated with cell differentiation. AML cell surface markers (such as CD33, CD123) serve as diagnostic and therapeutic targets. This is closely associated with plasma membrane.

Differential mRNA expressions of AML are concentrated in the KEGG cancer-related pathways of Ras signaling pathway, PI3K-Akt signaling pathway, NF-kappa B signaling pathway, MAPK signaling pathway, JAK-STAT signaling

pathway and so on (Supplementary Figure S17B). For example, the PI3K-Akt signalling pathway exhibits constitutive activation in over 50% of AML samples, promoting cell survival, inhibiting apoptosis, and being closely associated with drug resistance; its downstream mTOR pathway also represents a significant therapeutic target. The focal adhesion pathway mediates AML cell adhesion to stromal cells via integrins and cadherins, enhancing survival signals and shielding against chemotherapeutic agents. It constitutes the core structural basis for microenvironment-dependent drug resistance.

We also explored the signalling pathways potentially involved in differentially expressed miRNAs on AML using predicted targets from experimentally validated miRNA interactions within the DIANA-TarBase database. Supplementary Figure S18 shows that differentially expressed miRNAs participate in several pathways associated wit AML occurrence, proliferation, invasion, and metastasis including TGF-beta signaling pathway, p53 signaling pathway, Wnt signaling pathway, PI3K-Akt signaling pathway, MAPK signaling pathway, mTOR signaling pathway and so on. Moreover, miR-326 has been demonstrated to be associated with AML [10].

Evaluation of subtypes identified in COAD. Similarly, we performed differential expression analysis with mRNA and miRNA data on COAD to explore whether the identified subtypes identified by seOMLR possess biological significance.

GO terms and KEGG pathway enrichment analysis were both conducted on differentially expressed mRNAs of COAD (Supplementary Figure S19). Cancer-related GO semantic terms on COAD contain key functional modules such as cell adhesion, cell differentiation, inflammatory response, negative regulation of cell population proliferation, plasma membrane, collagen-containing extracellular matrix, integrin binding and so on (Supplementary Figure S19A), which play pivotal roles in malignant tumours initiation, progression, metastasis, and therapy. In the biological process of negative regulation of cell population proliferation, the APC gene inactivation leads to $\beta-$catenin accumulation, thereby relieving negative control over the cell cycle. This represents the most common driver mutation in COAD. Certain differential mRNAs of COAD exhibited concentrated enrichment in cancer-related KEGG pathways of Wnt signaling pathway, PI3K-Akt signaling pathway, focal adhesion, cell adhesion molecules, Ras signaling pathway, MAPK signaling pathway, drug metabolism - cytochrome P450, cAMP signaling pathway, metabolism of xenobiotics by cytochrome P450 and so on (Supplementary Figure S19B). For instance, the Wnt signalling pathway serves as the molecular engine driving the initiation and progression of COAD, and remains the primary focus of current targeted therapies.

Furthermore, we also explored the signalling pathways potentially involved in differentially expressed miRNAs on COAD using predicted targets from experimentally validated miRNA interactions within the DIANA-TarBase database. These differentially expressed miRNAs on COAD participate in several pathways associated with cancer occurrence, proliferation, invasion, and metastasis including p53 signaling pathway, mTOR signaling pathway, Wnt signaling pathway, PI3K-Akt signaling pathway, MAPK signaling pathway and so on, shown

in Supplementary Figure S20. The TP53 gene is a crucial tumor suppressor gene closely associated with the p53 signalling pathway. The mutation of this gene results in loss of p53 protein function, preventing cells from undergoing normal apoptosis or repairing DNA damage, thereby promoting tumour growth and metastasis. Approximately 50% of colorectal cancers exhibit p53 mutations, leading to dysregulation of cell cycle and apoptosis control. Besides, miR-326 has been demonstrated to be associated with COAD [11].

Experiments across various datasets indicate the applicability of seOMLR and the biological significance of the identified subtypes.

References

[1] Song W, Wang W, Dai D. Subtype-WESLR: identifying cancer subtype with weighted ensemble sparse latent representation of multi-view data. *Briefings in Bioinformatics*, 23(1):bbab398, 2022.

[2] Nie F, Li J, Li X. Parameter-free auto-weighted multiple graph learning: A framework for multiview clustering and semi-supervised classification. *Proceedings of the Twenty-Fifth International Joint Conference on Artificial Intelligence: IJCAI-16, New York City, New York, USA*, 9-15 July 2016, Volume Three, 2016.

[3] Lin Z, Liu R, Su Z. Linearized Alternating Direction Method with Adaptive Penalty for Low-Rank Representation. *Advances in Neural Information Processing Systems*, pages 612–620, 2011.

[4] Guo X, Wang X, Ling H. Exclusivity Regularized Machine: A New Ensemble SVM Classifier. *Proceedings of the Twenty-Sixth International Joint Conference on Artificial Intelligence*, 1739-1745, 2017.

[5] Huang J, Nie F, Huang H. Spectral rotation versus K-Means in spectral clustering. *Proceedings of the Twenty-Seventh AAAI Conference on Artificial Intelligence*, pages 431-437, 2013.

[6] Shimomura A, Shiino S, Kawauchi J, et al. Novel combination of serum microRNA for detecting breast cancer in the early stage. *Cancer Science*, 107(3):326-334, 2016.

[7] Nakagawa T, Kollmeyer T M, Morlan B W, et al. A tissue biomarker panel predicting systemic progression after PSA recurrence post-definitive prostate cancer therapy. *PLoS One*, 3(5):e2318, 2008.

[8] Conway K, Edmiston S N, Tse C K, et al. Racial variation in breast tumor promoter methylation in the Carolina Breast Cancer Study. *Cancer Epidemiology and Prevention Biomarkers*, 24(6):921-930, 2015.

[9] Shi Q, Zhang C, Peng M, et al. Pattern fusion analysis by adaptive alignment of multiple heterogeneous omics data. *Bioinformatics*, 33(17):2706-2714, 2017.

[10] Saffari N, Rahgozar S, Faraji E, et al. Plasma-derived exosomal miR-326, a prognostic biomarker and novel candidate for treatment of drug resistant pediatric acute lymphoblastic leukemia. *Scientific Reports*, 14(1):1-13, 2026.

[11] Wang Z, Jiang X, Li Q, et al. Integrated analysis identifies low microRNA-215 expression as associated with a poor prognosis of patients with colorectal cancer through the IKβ-α signaling pathway. *Translational cancer research*, 9(9):5233-5244, 2020.

Supplementary Table

Table S1: An Overview of eight cancer cohorts from TCGA

Cancer type	KIRC	BRCA	COAD	SKCM	GBM	LUSC	AML	SARC
miRNA	329	354	350	395	534	396	246	333
mRNA	16599	16699	16373	15955	12042	16841	14970	16026
DNA methylation	5000	5000	5000	5000	5000	5000	5000	5000
size	206	623	214	439	271	337	159	261

Table S2: Survival analysis of distinct methods on TCGA data. Values of C_index of Log-rank test are used for statistical significance test. Best results are in boldface.

Cancer type	KIRC	BRCA	COAD	SKCM	GBM	LUSC	AML	SARC
NEMO	0.654	0.526	0.557	0.560	0.533	0.565	0.569	0.599
iClusterPlus	0.570	0.589	0.572	0.521	0.529	0.530	0.587	0.585
iClusterBayes	0.617	0.535	0.552	0.542	0.515	0.516	0.554	0.58
moCluster	0.626	0.588	0.543	0.566	0.538	0.576	0.593	0.585
MDICC	0.553	0.550	0.610	0.569	0.524	0.541	0.567	0.562
SNF	0.638	0.587	0.568	0.565	0.544	0.566	0.567	0.605
PartIES	0.549	0.584	0.621	0.534	0.520	0.558	0.563	0.600
k-means(all)	0.603	0.592	0.520	0.544	0.535	0.527	0.585	0.594
subtype-WESLR	0.660	0.595	0.632	0.580	0.559	0.587	0.603	0.606
seOMLR	**0.674**	**0.647**	**0.662**	**0.608**	**0.586**	**0.598**	**0.640**	**0.646**

Table S3: Comparison between the resulted subtypes by seOMLR and the previously reported subtypes on GBM based on mRNA and DNA methylation data, respectively. Within the 271 samples on GBM, 266 belonged to known CIMP subtype categories, while 269 samples fell into known mRNA data subtype categories. The number in brackets denotes the number of each identified subtype by seOMLR.

	**Subtypes based on mRNA data**				**Subtypes based on methylation data**	
Subtype	Mesenchymal	Classical	Proneural	Neural	Non-G-CIMP	G-CIMP
subtype 1 (32)	20	3	3	5	31	0
subtype 2 (28)	8	8	9	2	21	4
subtype 3 (43)	6	2	28	7	20	23
subtype 4 (48)	8	28	4	8	47	0
subtype 5 (56)	12	15	16	13	55	1
subtype 6 (64)	29	12	12	11	64	0
Total	83	68	72	46	238	28

Table S4: Comparison between the resulted subtypes by seOMLR and the previously reported subtypes on BRCA based on molecular typing of PAM50 RNAseq. Of the 623 samples on BRCA, there are 383 known subtype categories. The number before slash in brackets denotes the number of known subtypes in the identified sutypes, and the number after slash in brackets indicates sample size of the identified sutypes.

PAM50RNAseq	Lum-A	Lum-B	Her2-enriched	Basal	Normal-like
total(383)	188	99	25	57	14
subtype 1(32/71)	1	0	1	28	2
subtype 2(59/73)	30	11	4	5	9
subtype 3(53/199)	32	17	2	0	2
subtype 4(21/22)	5	9	1	6	0
subtype 5(176/202)	111	58	7	0	0
subtype 6(31/44)	0	3	9	18	1
subtype 7(11/12)	9	1	1	0	0

Table S5: Comparison between the resulted subtypes by seOMLR and the previously reported subtypes on BRCA based on molecular characteristics.

Subtype	ER(+)	ER(-)	PR(+)	PR(-)	Her2(+)	Her2(-)
subtype 1	9	58	7	60	1	25
subtype 2	53	15	48	19	6	39
subtype 3	180	14	159	35	4	25
subtype 4	12	6	11	6	2	17
subtype 5	179	8	161	25	26	132
subtype 6	9	31	5	35	6	21
subtype 7	8	0	8	0	0	7

Supplementary Figure

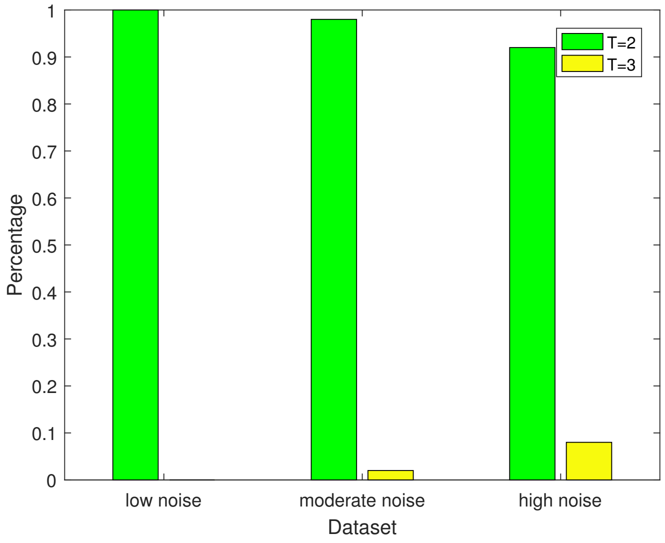

Figure S1: The bar chart about percentage of iterations on simulated dataset. We repeated 50 times on synthetic dataset under distinct noise and counted the number of iterations when the algorithm converges, and calculated the percentage of different iterations in 50 repeated experiments. We set maximum number of iterations to be 10. We can observe that the proposed seOMLR method generally converges within $2 \sim 3$ iterations, even under varying noise levels, and set the maximum iterations as $T = 10$ on simulated data.

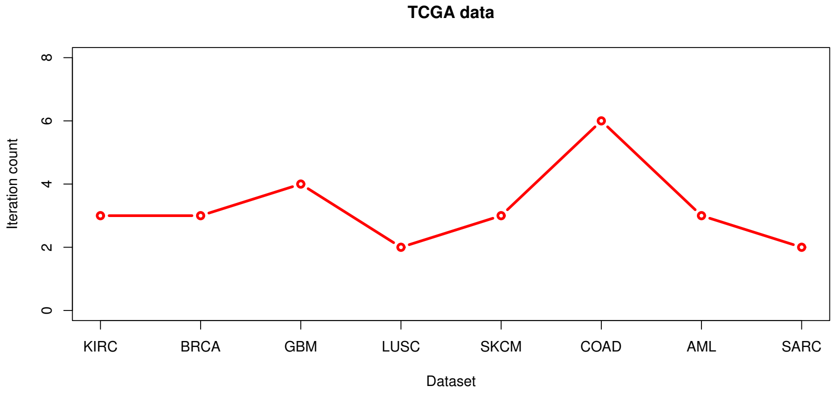

Figure S2: The curve of iteration times during convergence on TCGA data. We can observe that the proposed seOMLR method converges in $2 \sim 6$ iterations on average and set the maximum iterations as $T = 10$ on TCGA data.

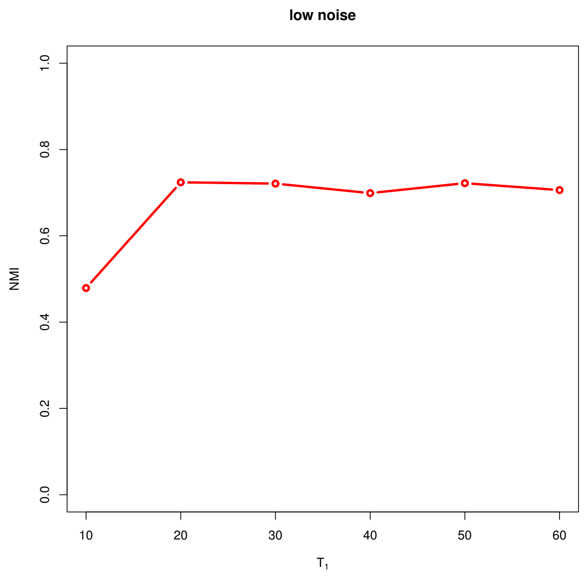

A

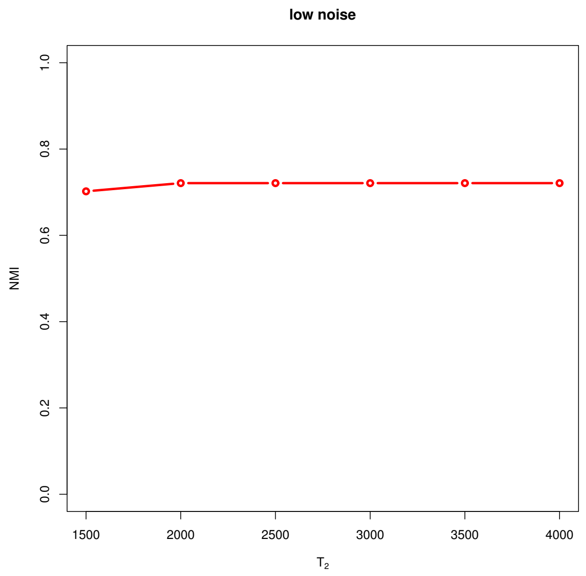

B

Figure S3: The sensitivity test of parameters T_1 and T_2 on simulated data under low noise. We set $T_1 = 30$ and $T_2 = 3000$.

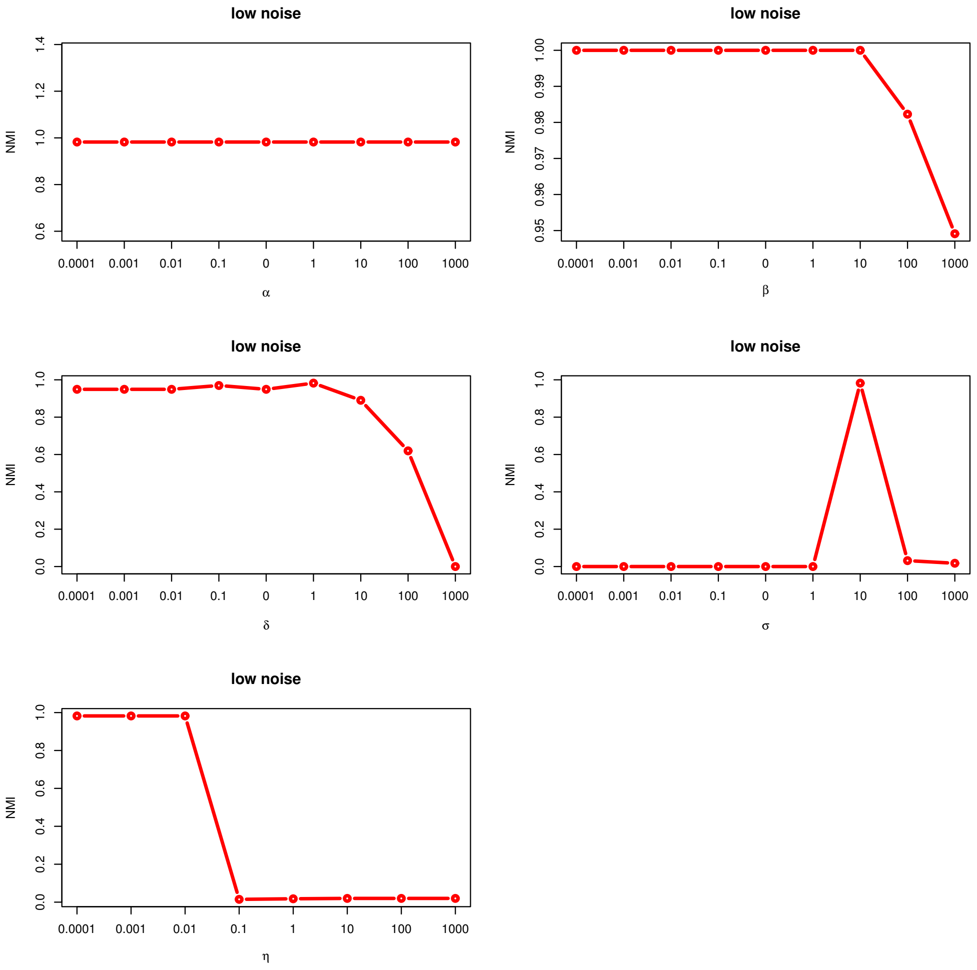

Figure S4: The sensitivity test of parameters α, β, δ, σ, and η on simulated data under low noise. We set $\alpha = 1$, $\beta = 100$, $\delta = 1$, $\sigma = 10$, and $\eta = 0.001$.

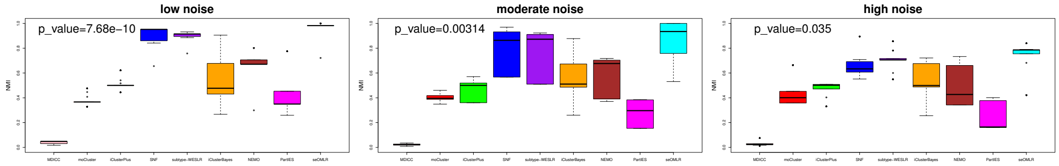

A

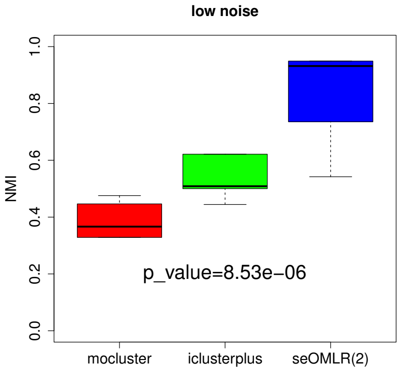

B

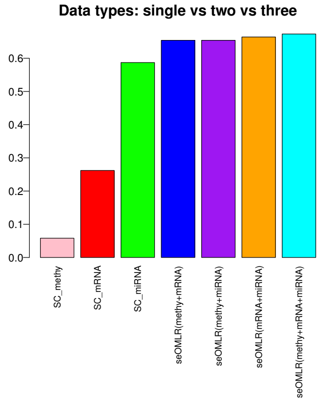

C

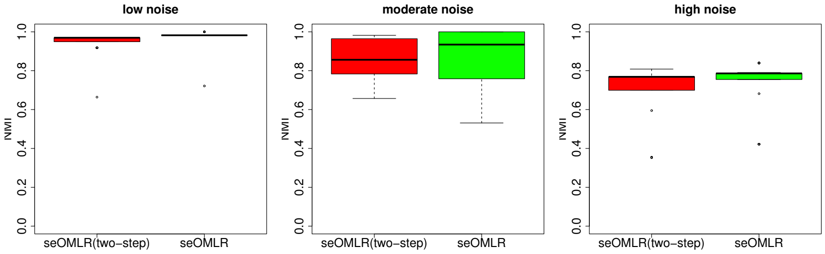

D

Figure S5: Analysis on simulated dataset. (A) Values of NMI among MDICC, moCluster, iClusterPlus, SNF, subtype-WESLR, iClusterBayes, NEMO, PartIES, and seOMLR under distinct noise including low, moderate, and high noise, which measure the concordance between the obtained clustering and the ground-truth. $p\_value = 7.68 \times 10^{-10}$, $p\_value = 0.00314$, and $p\_value = 0.035$ indicate the significant difference between SNF and enECMSC under distinct extra noise by two sample t-test. (B) Values of NMI among moCluster, iClusterPlus, and seOMLR(2) using moCluster and iClusterPlus as base methods. $p\_value = 8.53 \times 10^{-6}$ indicates the significant difference between iClusterPlus and seOMLR(2) by two sample t-test. (C) Values of NMI among SC-methylation, SC-miRNA, SC-mRNA, seOMLR(mRNA+miRNA), seOMLR(methy+miRNA), seOMLR(methy+mRNA), and seOMLR(methy+mRNA+miRNA). We applied seOMLR to data by combining any two of DNA methylation, miRNA, and mRNA, named as seOMLR(mRNA+miRNA), seOMLR(methy+miRNA), and seOMLR(methy+mRNA), respectively. In seOMLR(mRNA+miRNA), SC-miRNA and SC-mRNA were used as base methods. SeOMLR(methy+miRNA) and seOMLR(methy+mRNA) take similar approach, and seOMLR(methy+mRNA+miRNA) employs SC-mRNA, SC-methylation, and SC-miRNA as base methods. (D) Values of NMI between seOMLR and seOMLR(two-step) under distinct noise. SeOMLR(two-step) is a variant where seOMLR stops at the continuous representation matrix F and uses k-means for discretization.

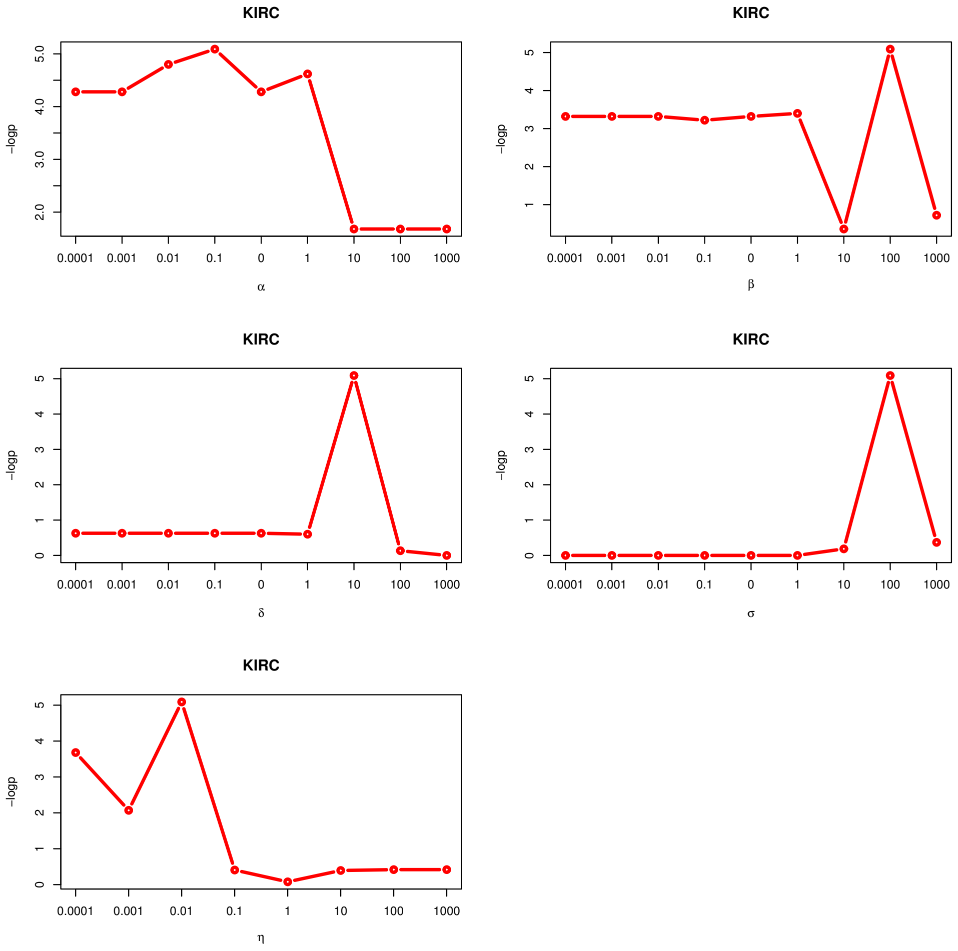

Figure S6: The sensitivity test of parameters α, β, δ, σ, and η on KIRC. We set $\alpha = 0.1$, $\beta = 100$, $\delta = 10$, $\sigma = 100$, and $\eta = 0.01$.

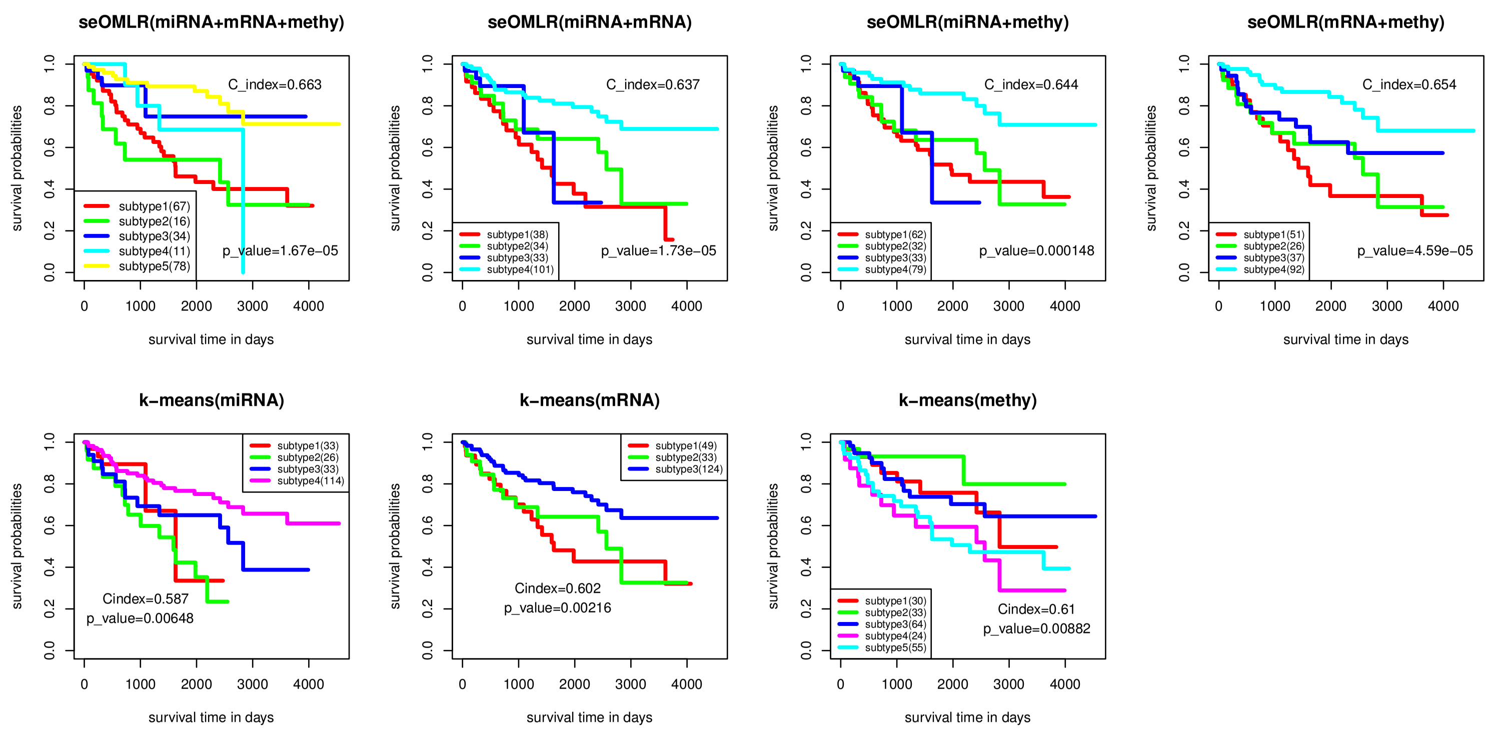

Figure S7: Kaplan-Meier survival curves of seOMLR(miRNA+mRNA+methylation), seOMLR(miRNA+mRNA), seOMLR(miRNA+methy), seOMLR(mRNA+methy), k-means(miRNA), k-means(mRNA), and k-means(methy) on KIRC. We performed k-means clustering on three KIRC data types, named as k-means(miRNA), k-means(mRNA), and k-means(methy), which are employed as inputs of seOMLR for ensemble learning, denoted as seOMLR(miRNA+mRNA+methy). Besides, we also explored multiple scenarios wherein the clustering outputs from k-means(miRNA), k-means(mRNA), and k-means(methy) were provided to seOMLR as inputs in any pairwise combination, named as seOMLR(miRNA+mRNA), seOMLR(miRNA+methy) and seOMLR(mRNA+methy). In seOMLR(miRNA+mRNA), k-means(miRNA) and k-means(mRNA) were used as base methods. SeOMLR(miRNA+methy) and seOMLR(mRNA+methy) take similar approach. The number in brackets of legend represents the number of member belonged to each subtype for cancers.

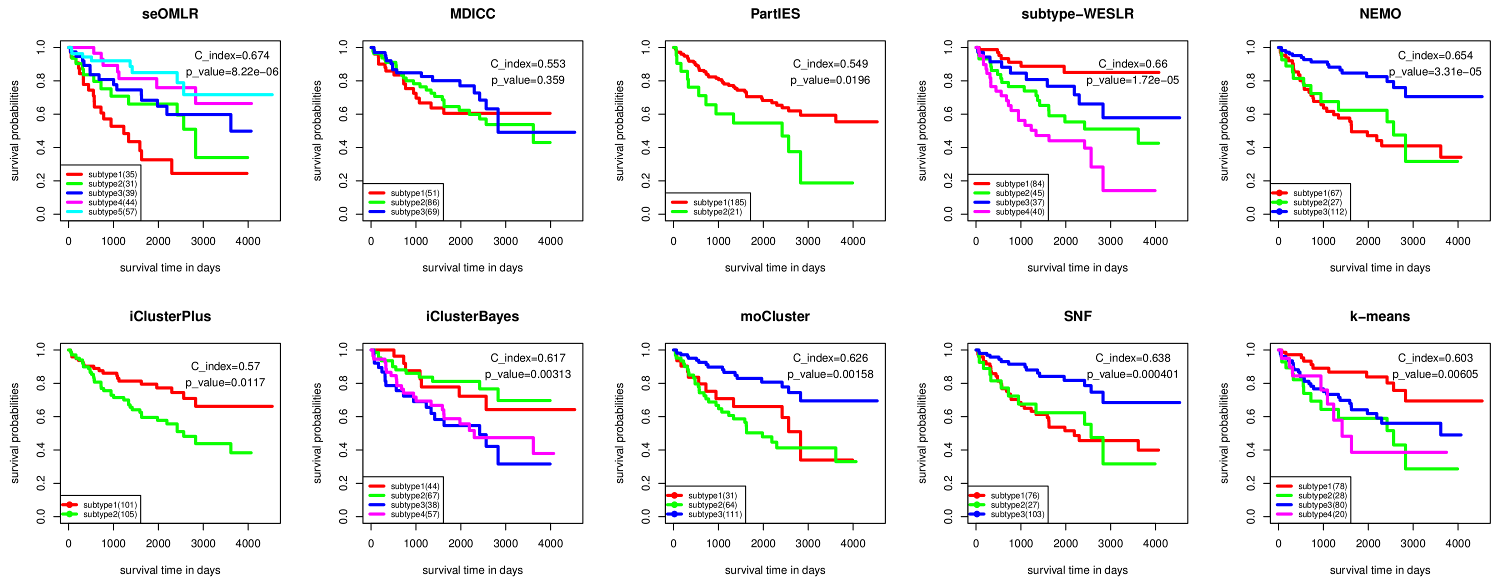

Figure S8: Kaplan-Meier survival curves of seOMLR, MDICC, PartIES, subtype-WESLR, NEMO, iClusterPlus, iClusterBayes, moCluster, SNF, and k-means on KIRC.

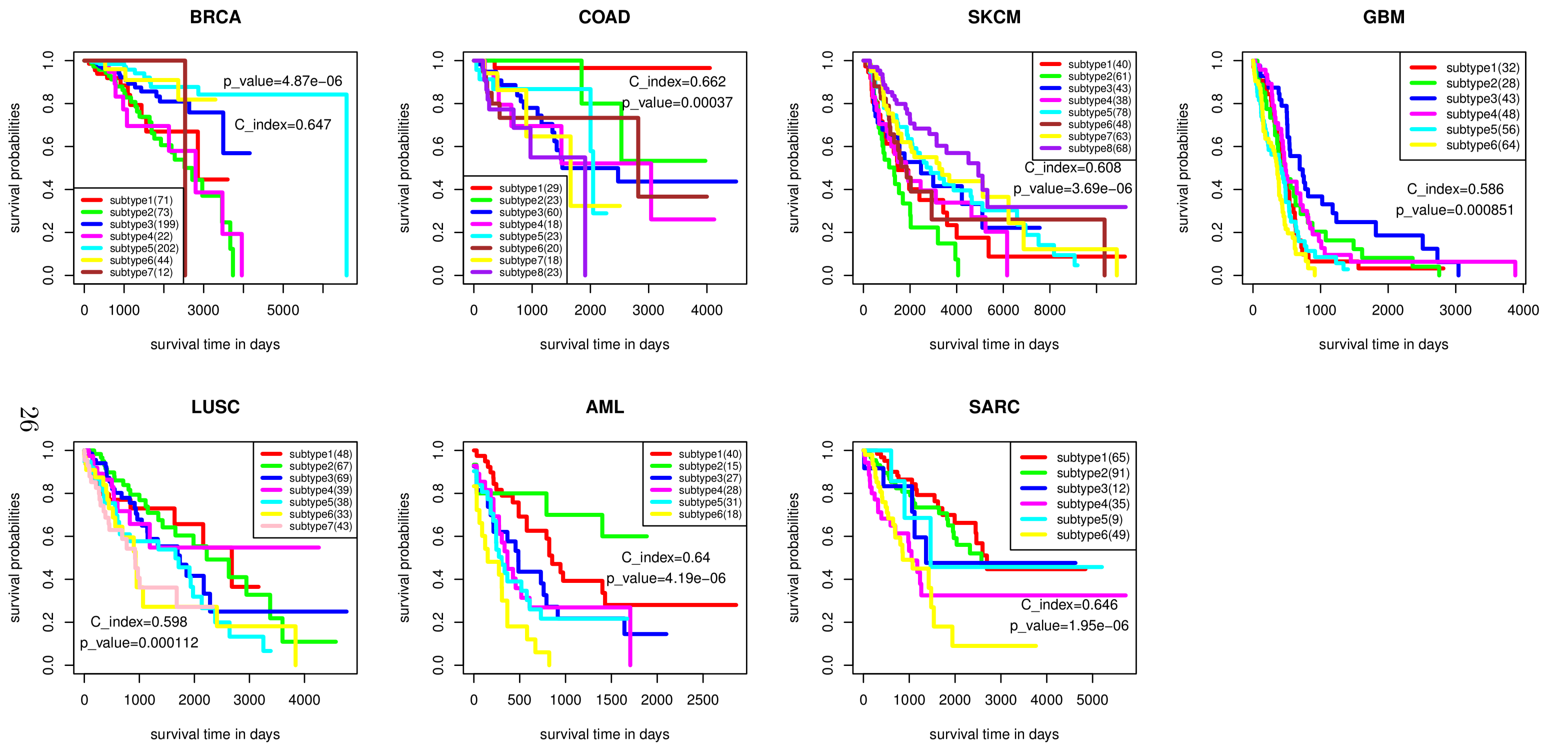

Figure S9: Kaplan-Meier survival curves of seven cancer cohorts including BRCA, COAD, SKCM, GBM, LUSC, AML, and SARC by seOMLR. The number in brackets of legend represents the number of member belonged to each subtype for cancers.

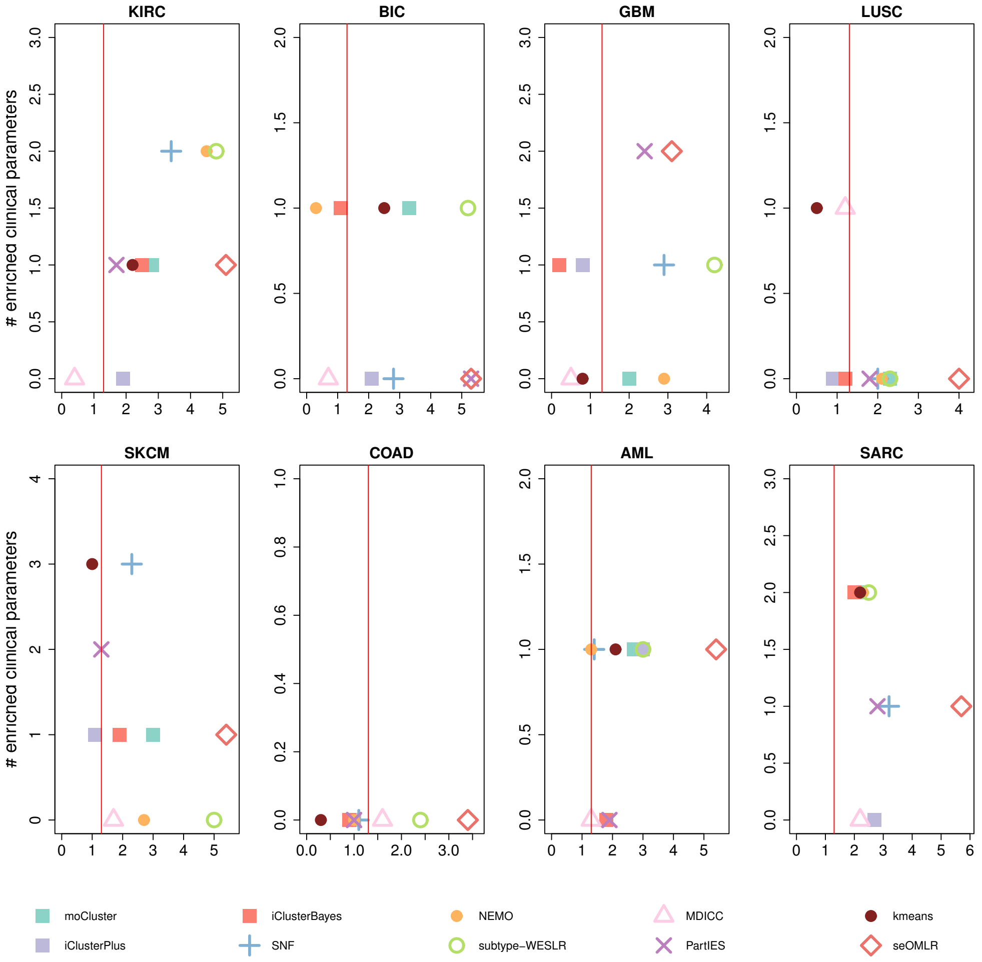

Figure S10: Enrichment of clinical labels including age, gender, pathological T, pathological M, pathological N, and pathological stage of eight cancer cohorts among distinct methods. The x-axis denotes the negative $log_{10}p$_value of the Log-rank test, while the y-axis represents the number of clinically enriched labels. Red vertical lines delineate the threshold for statistically significant survival differences (p_value ≤ 0.05).

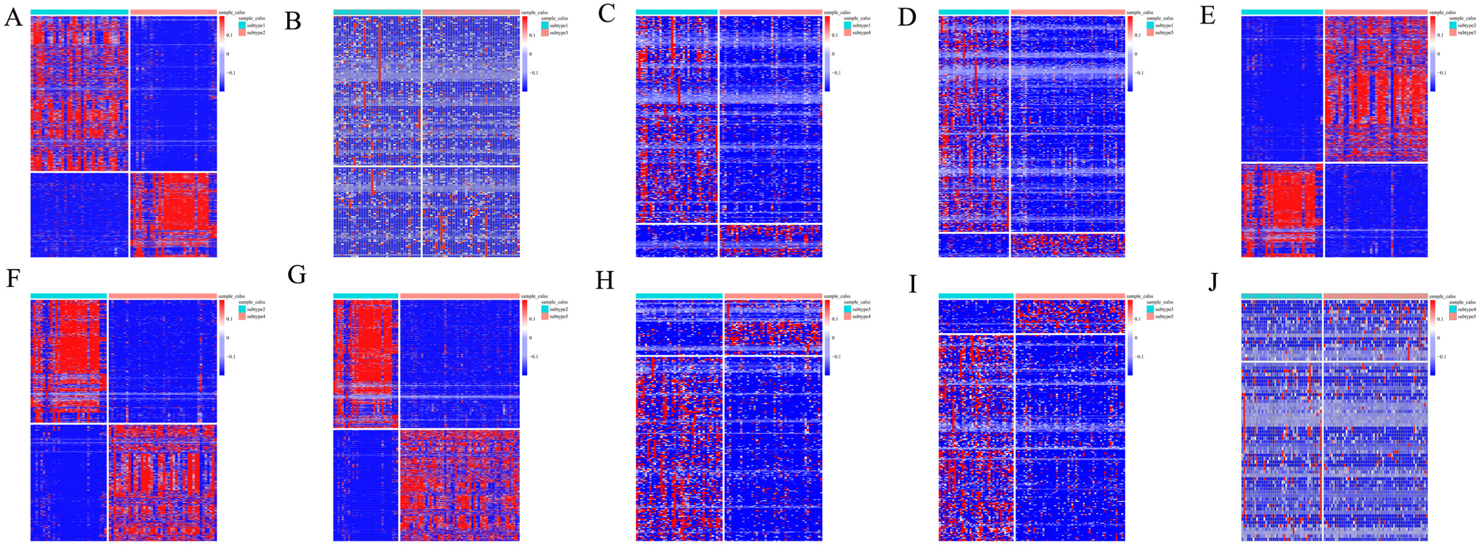

Figure S11: Heatmaps of significantly differential expressed mRNA among KIRC subtypes. (A) subtypes 1 and 2; (B) subtypes 1 and 3; (C) subtypes 1 and 4; (D) subtypes 1 and 5; (E) subtypes 2 and 3; (F) subtypes 2 and 4;(G) subtypes 2 and 5; (H) subtypes 3 and 4; (I) subtypes 3 and 5; (J) subtypes 4 and 5. Rows represent individual genomic features, while columns denote distinct samples.

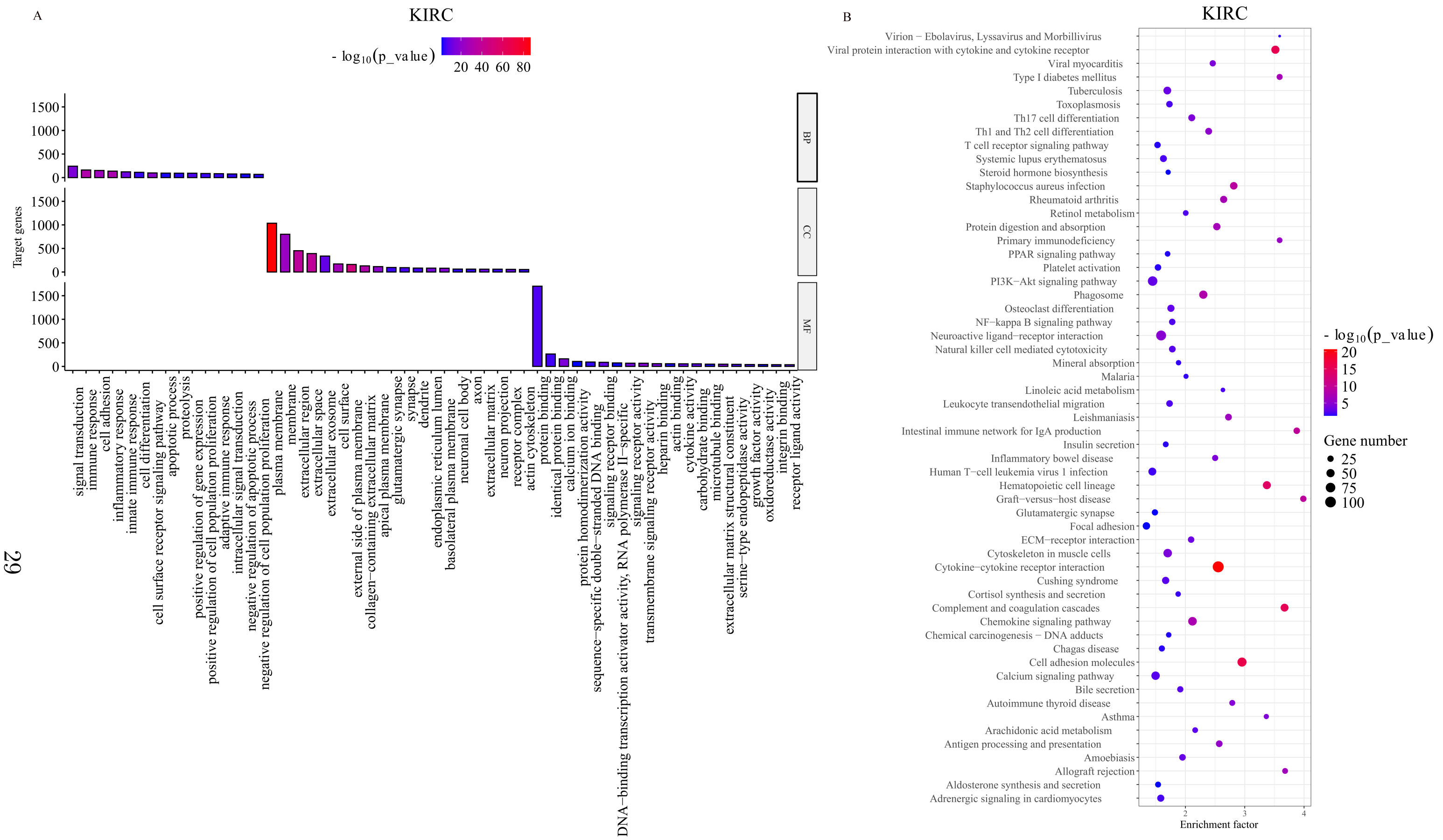

Figure S12: Enrichment analysis of differentially expressed mRNA on KIRC. (A) GO terms enrichment analysis. Cancer-related GO semantic terms encompass key functional modules such as cell adhesion, inflammatory response, cell differentiation, plasma membrane, cytokine activity and so on, which play pivotal roles in KIRC initiation, progression, metastasis, and therapy. For example, the cytokine activity pathway regulate immune responses and inflammation, influencing the tumour microenvironment. (B) KEGG signal pathway enrichment analysis. certain mRNAs exhibited concentrated enrichment in KEGG pathways like PI3K-Akt signaling pathway, NF-kappa B signaling pathway, Chemokine signaling pathway and so on, which are implicated in the malignant tumourigenesis, metabolism, invasion, and prognosis. For example, the NF-kappa B signalling pathway regulates inflammatory responses and cell survival, promotes the formation of the tumour microenvironment, and is associated with the progression and drug resistance of KIRC.

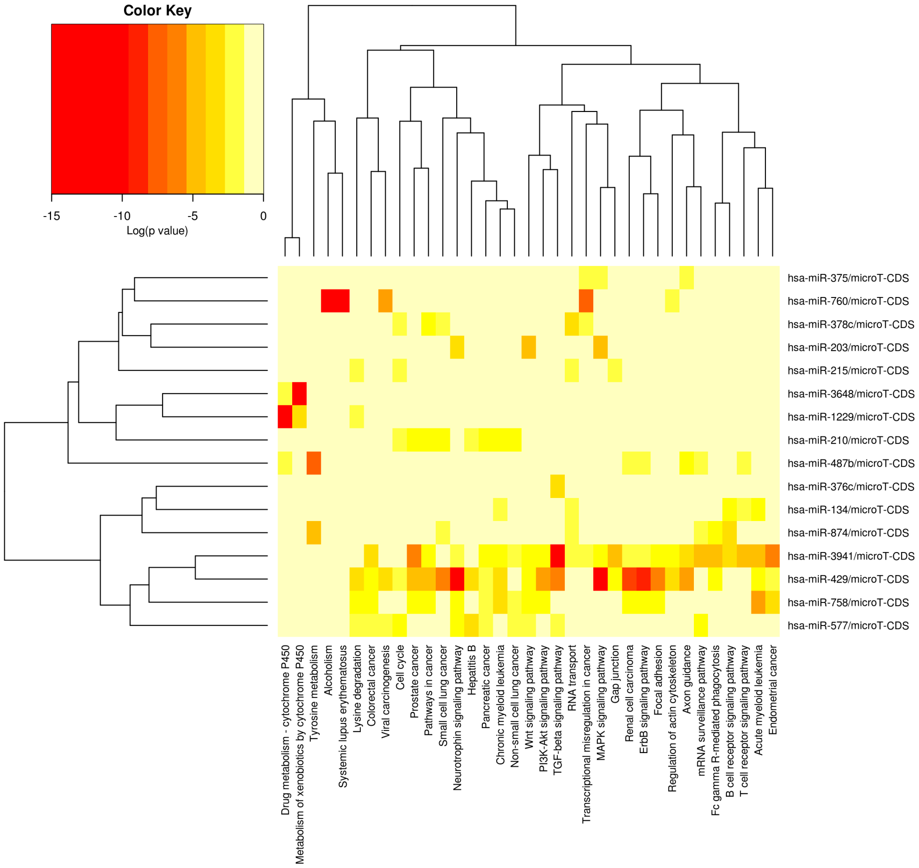

Figure S13: Significant signaling pathways about the differentially expressed miRNAs on KIRC by utilizing the DIANA-miRPath. These differentially expressed miRNAs participate in several pathways associated with tumourigenesis, progression, and metastasis including Wnt signaling pathway, PI3K-Akt signaling pathway, TGF-beta signaling pathway, MAPK signaling pathway, Transcriptional misregulation in cancer and so on.

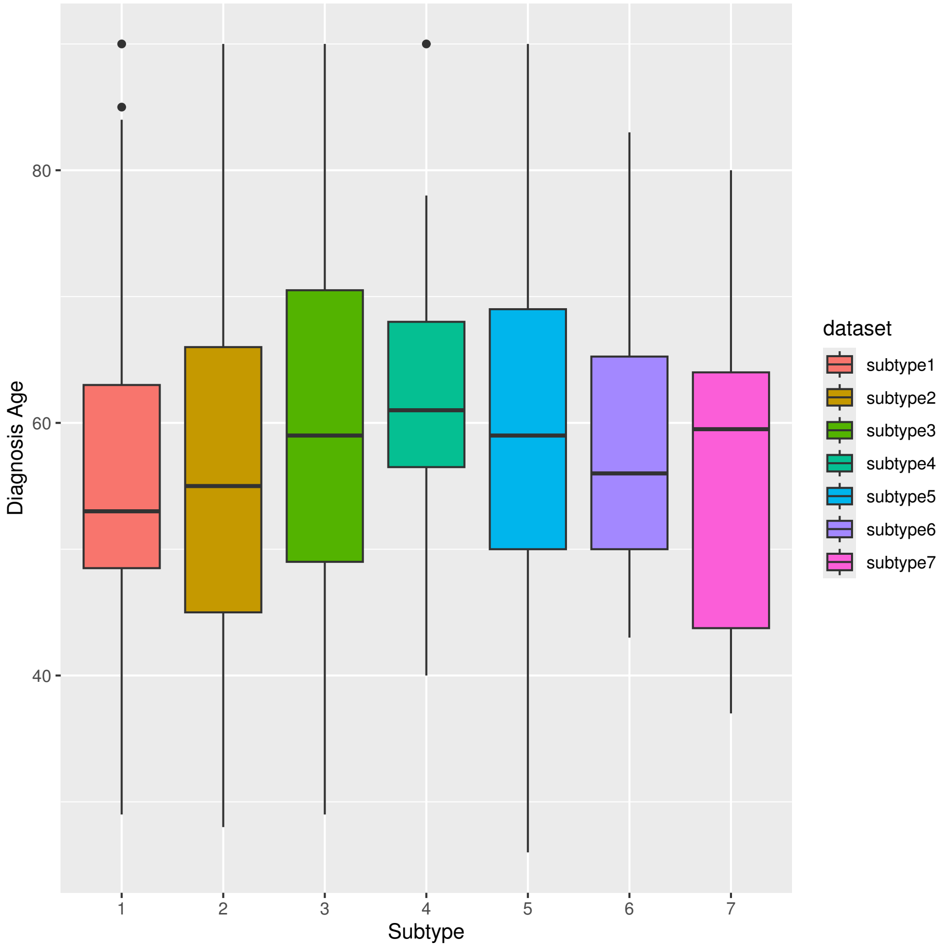

Figure S14: Diagnosis ages of the identified subtypes on BRCA.

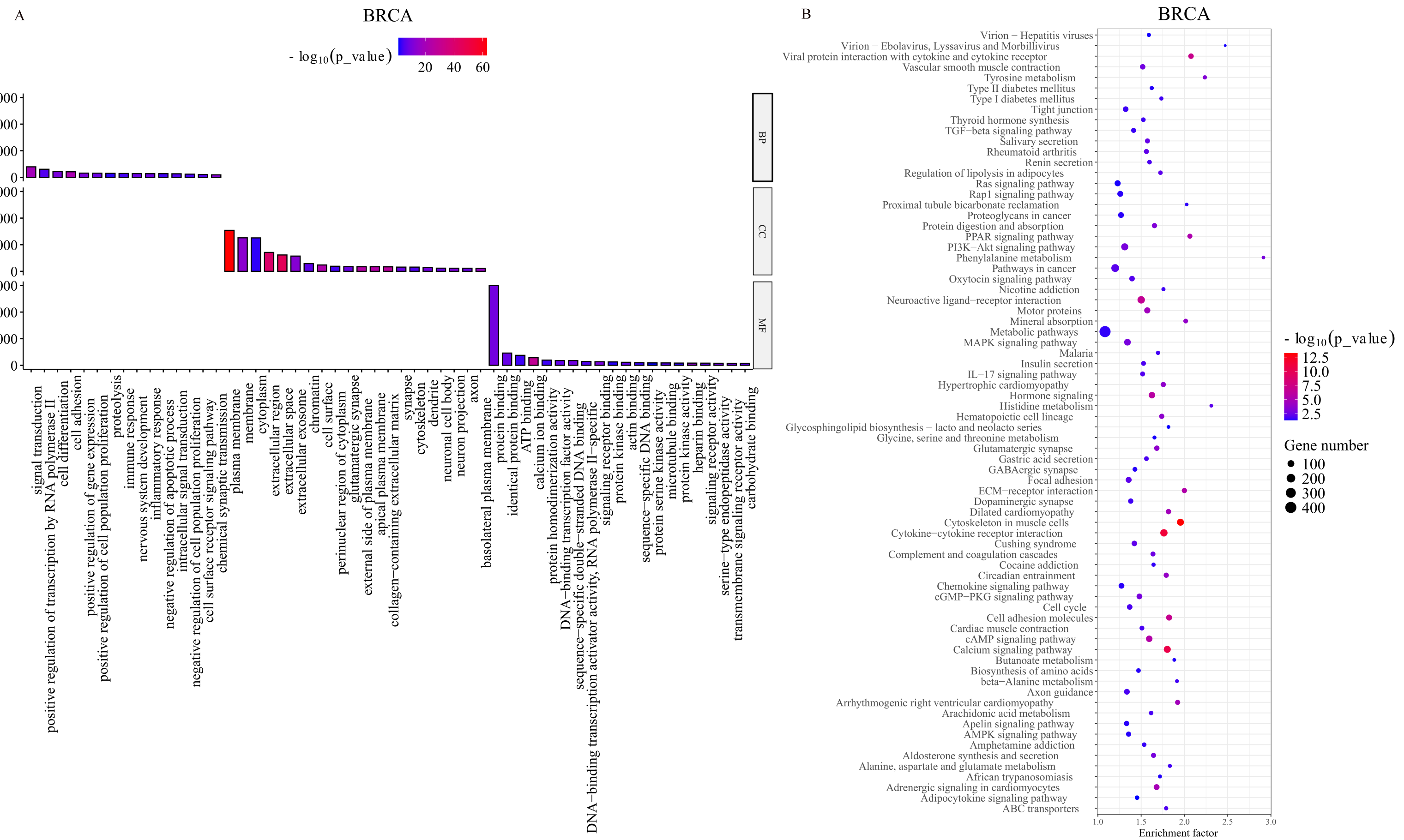

Figure S15: Enrichment analysis of differentially expressed mRNA on BRCA. (A) GO terms enrichment analysis; (B) KEGG signal pathway enrichment analysis.

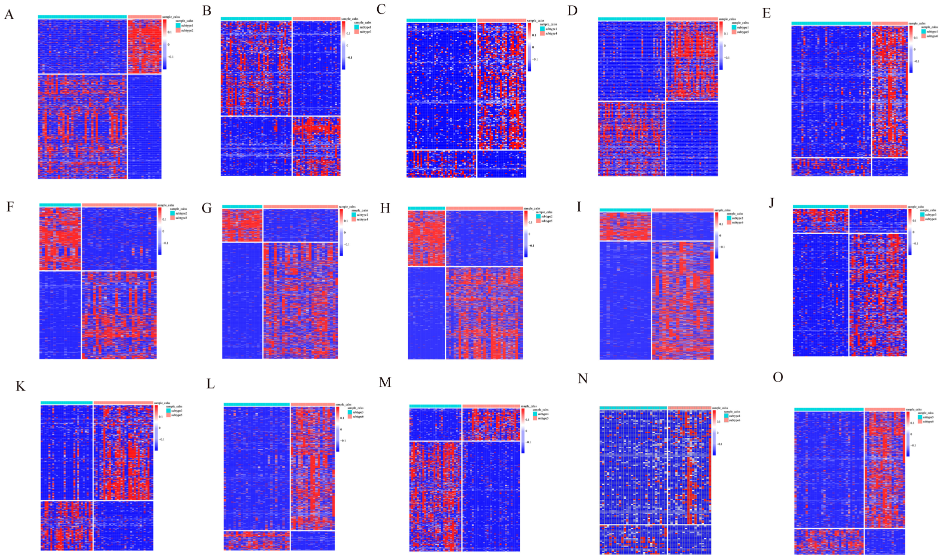

Figure S16: Heatmaps of significantly differential expressed mRNA among AML subtypes. (A) subtypes 1 and 2; (B) subtypes 1 and 3; (C) subtypes 1 and 4; (D) subtypes 1 and 5; (E) subtypes 1 and 6; (F) subtypes 2 and 3; (G) subtypes 2 and 4; (H) subtypes 2 and 5; (I) subtypes 2 and 6; (J) subtypes 3 and 4; (K) subtypes 3 and 5; (L) subtypes 3 and 6; (M) subtypes 4 and 5; (N) subtypes 4 and 6; (O) subtypes 5 and 6. Rows represent individual genomic features, while columns denote distinct samples.

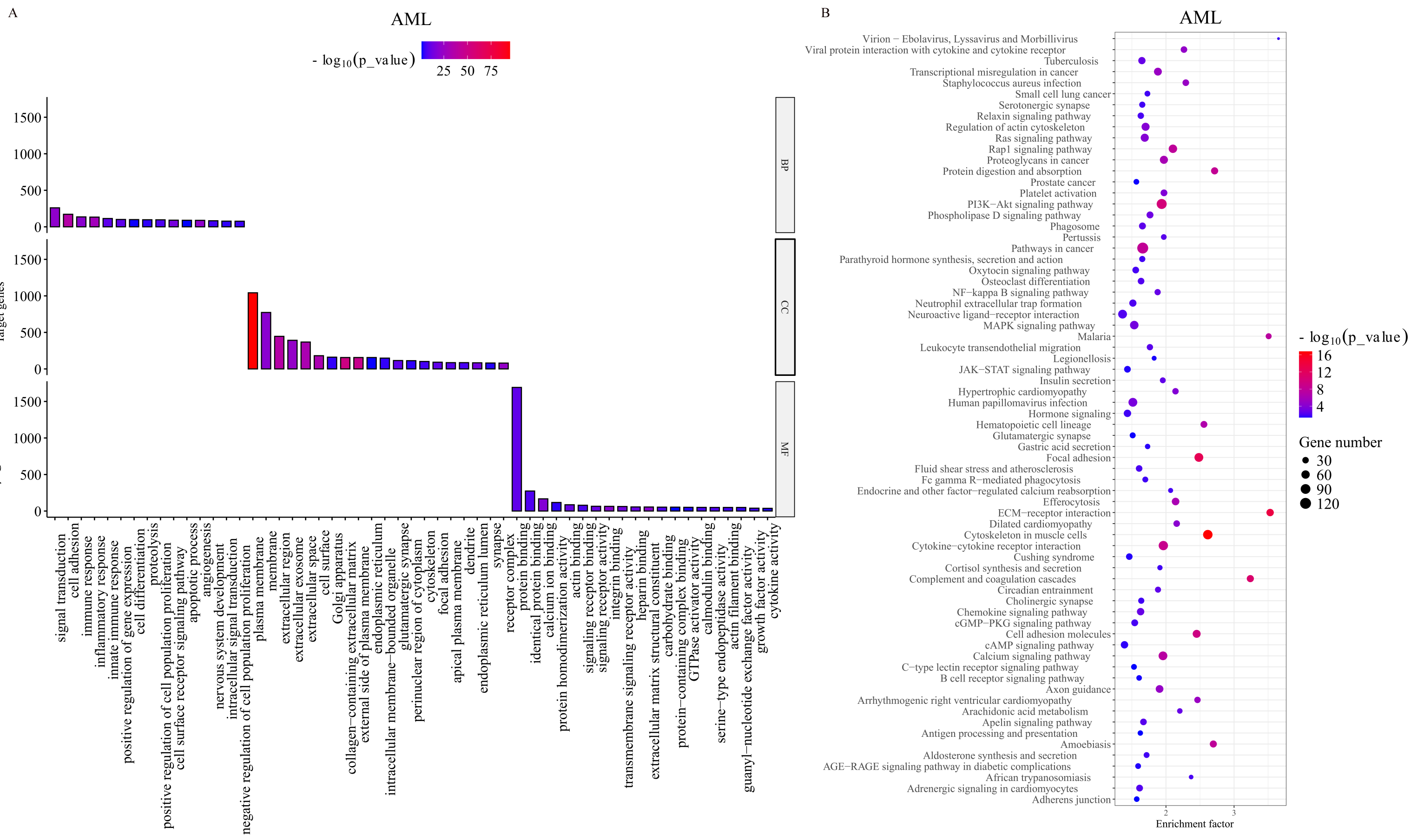

Figure S17: Enrichment analysis of differentially expressed mRNA on AML. (A) GO terms enrichment analysis; (B) KEGG signal pathway enrichment analysis.

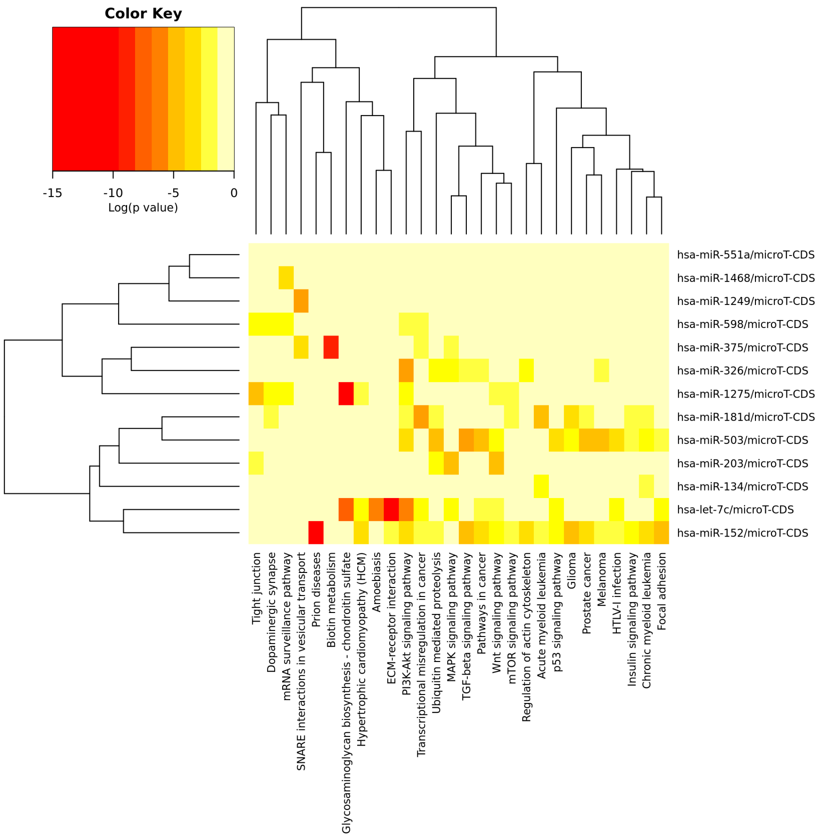

Figure S18: Significant signaling pathways about the differentially expressed miRNAs on AML by utilizing the DIANA-miRPath.

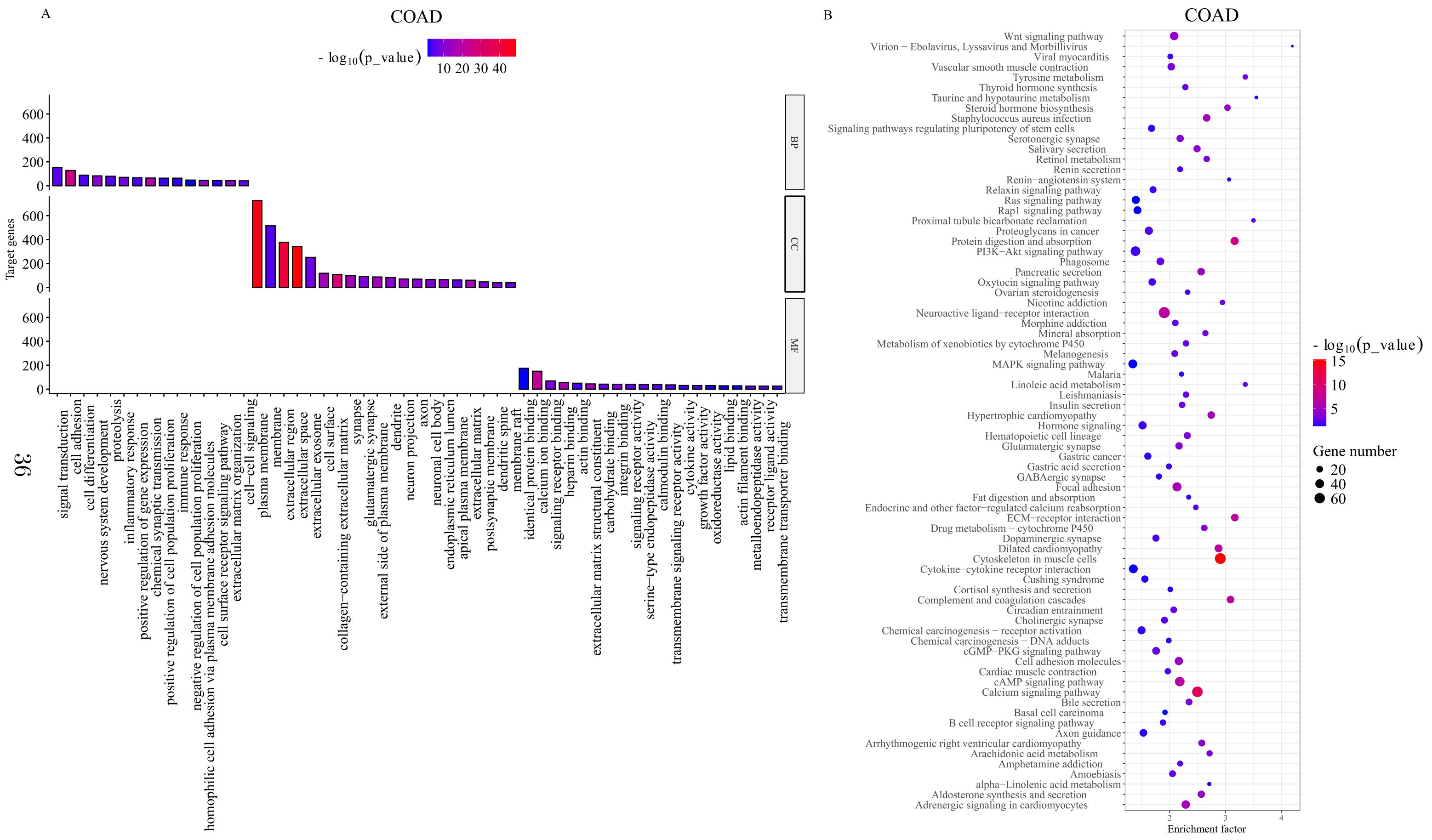

Figure S19: Enrichment analysis of differentially expressed mRNA on COAD. (A) GO terms enrichment analysis; (B) KEGG signal pathway enrichment analysis.

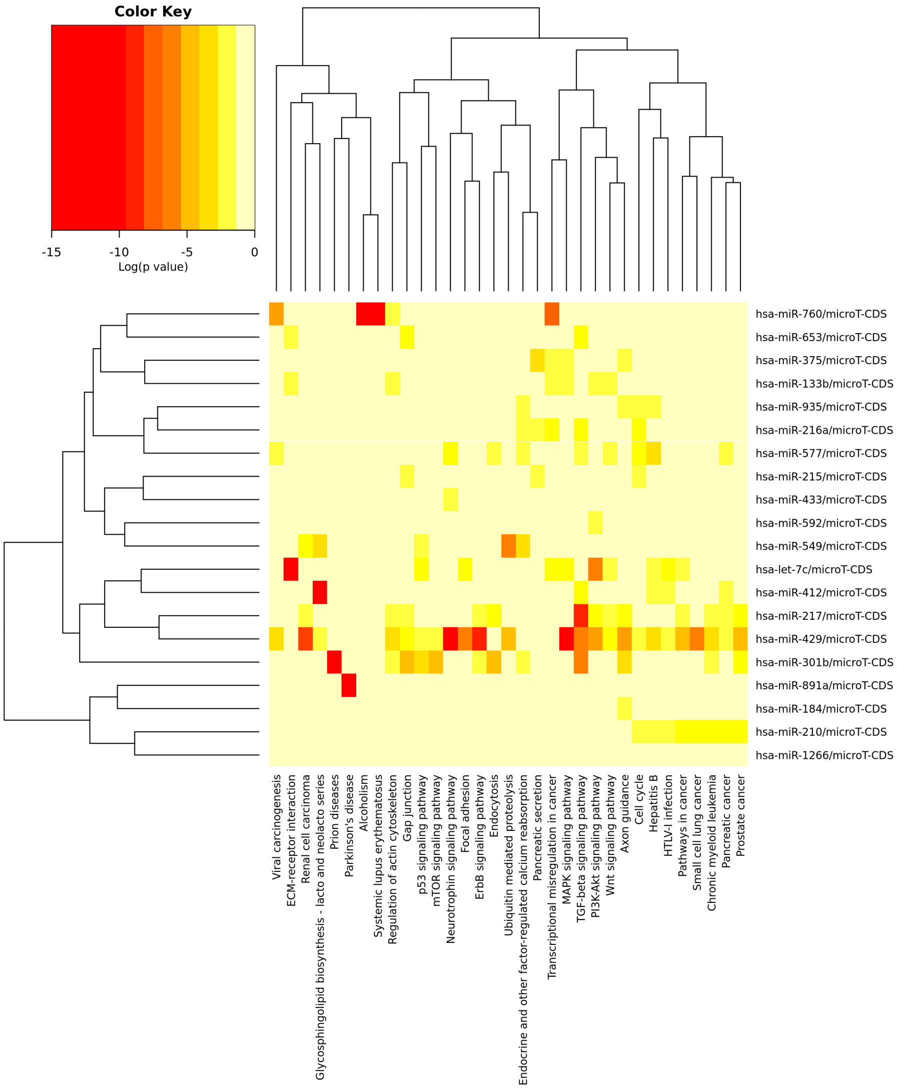

Figure S20: Significant signaling pathways about the differentially expressed miRNAs on COAD by utilizing the DIANA-miRPath.
